# Understanding the relative valuation of research impact: a best–worst scaling experiment of the general public and biomedical and health researchers

**DOI:** 10.1136/bmjopen-2015-010916

**Published:** 2016-08-18

**Authors:** Alexandra Pollitt, Dimitris Potoglou, Sunil Patil, Peter Burge, Susan Guthrie, Suzanne King, Steven Wooding, Jonathan Grant

**Affiliations:** 1 The Policy Institute at King's College London, London, UK; 2 School of Geography and Planning, Cardiff University, Cardiff, UK; 3 RAND Europe, Westbrook Centre, Cambridge, UK

**Keywords:** STATISTICS & RESEARCH METHODS, HEALTH ECONOMICS

## Abstract

**Objectives:**

(1) To test the use of best–worst scaling (BWS) experiments in valuing different types of biomedical and health research impact, and (2) to explore how different types of research impact are valued by different stakeholder groups.

**Design:**

Survey-based BWS experiment and discrete choice modelling.

**Setting:**

The UK.

**Participants:**

Current and recent UK Medical Research Council grant holders and a representative sample of the general public recruited from an online panel.

**Results:**

In relation to the study's 2 objectives: (1) we demonstrate the application of BWS methodology in the quantitative assessment and valuation of research impact. (2) The general public and researchers provided similar valuations for research impacts such as improved life expectancy, job creation and reduced health costs, but there was less agreement between the groups on other impacts, including commercial capacity development, training and dissemination.

**Conclusions:**

This is the second time that a discrete choice experiment has been used to assess how the general public and researchers value different types of research impact, and the first time that BWS has been used to elicit these choices. While the 2 groups value different research impacts in different ways, we note that where they agree, this is generally about matters that are seemingly more important and associated with wider social benefit, rather than impacts occurring within the research system. These findings are a first step in exploring how the beneficiaries and producers of research value different kinds of impact, an important consideration given the growing emphasis on funding and assessing research on the basis of (potential) impact. Future research should refine and replicate both the current study and that of Miller *et al* in other countries and disciplines.

**Strengths and limitations of this study**

- This study contributes to the evidence base on how different stakeholder groups (researchers and the general public) value different types of research impact, an area in which there is a lack of methodological and empirical research.
- This study is important because research funders are increasingly interested in measuring (and rewarding) the societal (or non-academic) impact of research.
- We demonstrate the first application of survey-based best worst scaling methodology in the quantitative assessment of research impact and show that the general public and researchers value research impacts in different ways.
- There are limitations related to the samples used, in that the general public sample was not fully representative of the population and the drop-out rate for the researcher sample was high.
- The conclusions should not be over-interpreted given the methodological nature of the research, including the complex mechanisms for eliciting valuations, and the fact that our methodology does not reveal reasons for the differences we observe. Further research in this area is recommended.

## Introduction

The assessment of the non-academic impact of research is not new,^1^ but there is a growing interest internationally in methodological approaches to identify and measure these research impacts.^2–6^ In the UK, research impact assessment was institutionalised through the 2014 Research Excellence Framework (REF), which included the review and grading of 6975 four-page impact case studies by researchers and research users.^7^ The results of REF are used to allocate around £1.6 billion annually of research funding to English Higher Education Institutes, 20% (or £320 million/year) of which is determined by impact beyond the research system, emphasising the need for robust, fair and transparent assessments of research impact.

There is strong evidence that research makes a significant contribution to society,^8–11^ and that contribution manifests itself in different ways.^12^ ^13^ For example, we know that the total economic return from biomedical and health research is between 24% and 28%,^9^ ^11^ and from analysis of the REF impact case studies that there are a wide variety of impact topics.^13^ These benefits might occur within the research system itself or more widely in areas such as healthcare, the environment, technology, the economy or culture. However, there remains a lack of methodological and empirical research on how the public value research impact^14^ ^15^ and how valuations may vary between stakeholder groups.^16^

To address this issue, we undertook online surveys with a representative sample of the UK public as well as current and recent Medical Research Council (MRC) grant holders to elicit their relative valuation of different types of research impact. Using a method known as ‘best–worst scaling’ (BWS),^17^ we asked survey participants to compare statements about different types of impact. An example of an impact statement would be: ‘research helps create new jobs across the UK’ or ‘research contributes to care being provided more cheaply without any change in quality’.

BWS is a preference elicitation method that helps understand how respondents choose or rank ‘best’ and ‘worst’ items in a list.^17^ In recent years, the method has gained popularity in health and social care as well as other disciplines.^18–20^ For example, in the development of the Adult Social Care Outcome Toolkit (ASCOT) measure, BWS was employed to establish preference weights for social care-related quality of life domains.^18^ Likewise, Coast *et al*^20^ used BWS to develop an index of capability for older people, focused on quality of life, with an intended use in decision-making across health and social care in the UK.

The results of the BWS survey were used to develop a model that elicited the perceived value of different types of research impact for different groups and segments of survey respondents, including whether the public have different valuations from researchers. This matters as the scientific community and research users are increasingly asked to assess potential and actual research impact as part of assessment processes, but have no empirical basis on which to value different types of impact.

To the best of our knowledge, this is the second time this type of analysis has been undertaken. The first study, funded by Canada's national health research agency, was a cross-sectional, national survey of basic biomedical researchers and a representative sample of Canadian citizens. The survey assessed preferences for research outcomes across five attributes using a discrete choice experiment.^16^ The authors concluded that citizens and researchers fundamentally prioritised the same outcomes for basic biomedical research. Notably, they prioritised traditional scientific outcomes and devalued the pursuit of economic returns.

The specific objectives of the current study are to:

1. Contribute methodologically to the assessment of research impact, by adapting BWS to the analysis of the relative valuations of research impact; and
2. Develop our understanding of how different types of research impact are valued by different stakeholder groups.

Below we provide details of the study population and method. In the Results section, we describe the characteristics of those who responded to the survey and present the best fit BWS models for health and biomedical researchers and the general public. In the conclusion we explore the strengths and limitations of our approach, and draw out key observations from our analysis.

## Methods

**Figure 1:**
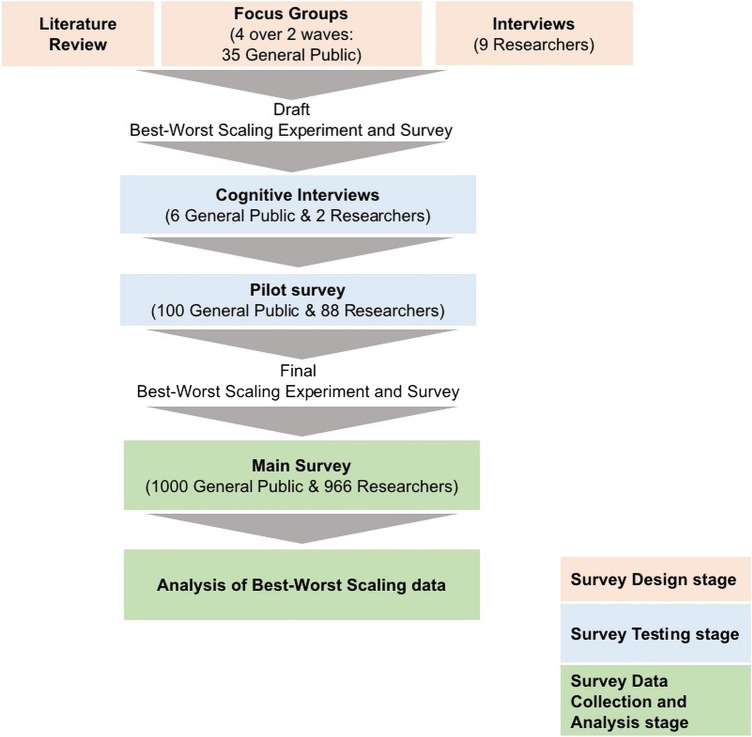
Overview of methods.

In this section, we describe: the BWS method; the study population; the two main stages of developing the survey instrument (defining and categorising the impacts of health and biomedical research, and constructing and testing the survey); the survey implementation and the data analysis carried out. Figure 1 provides an overview of the stages of the study.

### BWS method

We applied ‘attribute-level’ BWS,^21^ where we developed a set of lists that included different types (or ‘attributes’) of research impact each with varying degrees (or ‘levels’) of intensity. The respondents chose the ‘most important’ (best) and ‘least important’ (worst) impact from a series of lists. To elicit more information on the preference data, and to be able to more robustly determine the relative importance of the different types and degrees of research impact, we also asked respondents to choose the ‘second most important’ (second best) and ‘second least important’ (second worst) impacts from each list. Further details on the BWS method are provided in online supplementary file 1, while information on the modelling of respondents' data is provided under ‘data analysis’ below.

10.1136/bmjopen-2015-010916.supp1 Supplementary file

### Study population

The study focused on two populations—the general public and biomedical and health researchers—and different ‘segments’ or subpopulations within those populations. The general public participants were recruited from ResearchNow's internet panel. ResearchNow is a market research company that provides access to online panels of the public for surveys.^22^ We contracted ResearchNow for 1000 completed surveys based on representative quotas set on age, gender and regions in the UK. While no monetary incentive was offered to the researchers, the participants from the general public are paid by the market research agency for each survey they complete. For the general public, the questionnaire included a set of questions extracted from the Public Attitudes to Science 2014 study.^23^ These questions allowed individuals to be assigned to a segment based on their attitudes towards science.

The population of biomedical and health researchers was defined as all principal investigators who had held MRC funding between April 2006 and November 2014 (n=4708), regardless of whether the grant was still active, but excluding anyone who was not expected to enter grant information in Researchfish (a research evaluation data capture platform). The survey email invitations were sent to all 4708 researchers (except those used in the pilot). For researchers, the population segments were based on one of eight research activity code groups (eg, ‘underpinning research’, ‘health services research’, etc) from the Health Research Classification System (HRCS).^24^

### Defining and categorising the impacts of biomedical and health research

In this section, we describe the development of the content of the survey— that is, various kinds of impacts and ways of categorising them. This involved a literature review, focus groups and researcher interviews. The development phase is summarised here, with additional details provided in online supplementary file 2.

10.1136/bmjopen-2015-010916.supp2 Supplementary file

#### Literature review

We reviewed the literature with the aim of: (1) identifying a range of potential impacts of research; (2) investigating different ways of classifying impacts; and (3) producing a long list of possible categories and types of impact that could be tested in focus groups and interviews. The review largely covered grey literature as well as some academic literature and focused on a limited set of key sources known to the project team. It is summarised in online supplementary file 2. The main output of the review was a draft categorisation of research impacts for testing in the interviews and focus groups, as shown in table 1.

**Table 1:** Draft categorisation of impacts, developed from the existing literature

Category	Knowledge production and research targeting	Capacity building	Innovative and economic impact	Health and health sector benefit	Policy and public services (other than health)	Public engagement, dissemination, culture and creativity
Types of impacts and measures of those impacts included in this category	Volume and quality measures Future funding	Esteem measures Number and quality of researchers trained Collaboration and networking Wider participation in research	New products and process developed New businesses (spinouts) Benefits to companies Job creation, workforce development and increased economic competitiveness	Impact on guidelines/policy/professional training or development in health Impact on practice including saving NHS money Impact on health and well-being	Changes to policy outside of health Improvements in the delivery of public services (outside of health) Benefits to public well-being and society more widely	Number and range of dissemination and outreach activities Increased public understanding of and engagement with science

NHS, National Health Service.

#### Focus groups and researcher interviews

Focus groups with members of the public and interviews with individual researchers were used to refine the types of research impacts to be valued in the survey. Both methods aimed to identify the impacts the public and researchers expect to come from research, whether there is a shared understanding, how they could be categorised and how they might be measured.

Four focus groups over two waves were held with the general public. After an opening discussion about how to define biomedical and health research, the majority of the time in the groups was used to discuss types of impact, initially by asking participants to suggest ideas, then prompting discussion on any from our draft impact framework that had not been mentioned. For each item discussed, we aimed to determine if people considered it to be a possible impact of research, how it might be categorised (eg, health, economic, scientific) and how it might be measured. Details of the focus groups, including the topic guide, are provided in online supplementary file 2, and the key observations are summarised in box 1.

Box 1 **Key observations from the focus groups**

Definition of research

Following brainstorming on the evocation of the word ‘research’, participants agreed with our proposed definition that research is ‘studying something so that we (as humankind) can understand better how it works’. Health research and medical research were seen as slightly different, with health research considered as a broader term relating to research into health and lifestyle, understanding causes, and understanding ‘who suffers from what’. In contrast, medical research was considered as being more technical, focused on looking for cures and usually thought of as concerning drug development. We used the term ‘biomedical and health research’ in the final survey to encourage participants to think about a range of research.

Research impact

Research impacts from health and medical research suggested by participants were focused on better health, better quality of life and longevity. Hence, the purpose of medical research was generally seen as producing cures and ways of preventing illness and, to a lesser extent, improving palliative care. Most of the other impacts in our draft framework were also considered feasible once suggested by the facilitator.

Research process

Generally, little was known about research processes, infrastructure and practices, such as academic journal publications. This led to the exclusion of statements referring to technical or specialised aspects of the research process in the final survey (eg, different types of journal).

In addition to the focus groups, we undertook a small number of interviews (n=9) with biomedical and health researchers. Details of how the interviewees were selected, the interview protocol and the key observations are in online supplementary file 2. Interviewees were asked about their understanding of research impact, the kinds of impact that research in their field might have, and how these impacts might be categorised. We asked specifically about any items from our draft framework that were not mentioned unprompted by the interviewee.

When asked what they understood by the term ‘impact’ and to provide examples from their field, interviewees demonstrated a detailed and consistent awareness of research impact, particularly with respect to impact occurring outside the research system. Many referred to REF, and in conducting the interviews in the wake of this exercise, it is difficult to establish the extent to which this may have influenced perceptions of impact. Interviewees also referred to the research councils' increased emphasis on downstream impact, mentioning the need to outline potential benefits in funding applications. This is, perhaps, unsurprising given that interviewees were all MRC grant holders and were also aware that the present study was supported by the MRC. Researchers interviewed were broadly in agreement with the draft framework developed from the literature, both in terms of the overall domains representing a logical classification of impacts, and the specific items within them being impacts that could reasonably be expected to come from research. Five items in the draft framework were not considered by interviewees to be impacts and so were removed. These are detailed in online supplementary file 2.

### Constructing and testing the survey instrument

Findings from the literature review, focus groups and researcher interviews were used to inform the development of a list of attributes and levels for the BWS experiment, the key considerations being that the impacts used were both well understood and considered as feasible outcomes of biomedical and health research by both populations. The survey instrument was then tested through cognitive interviews and a pilot. Additional details of the survey construction and testing are provided in online supplementary file 3.

10.1136/bmjopen-2015-010916.supp3 Supplementary file

For the general public, the survey questionnaire included screening questions, an introduction to the BWS experiment and its tasks, questions relating to attitudes to science, and sociodemographic questions. Screening questions asked respondents about their age, gender, region of residence, social grade and work status. For researchers, the survey questionnaire included questions on research background, job title, clinical experience (if any), an introduction to the BWS experiment and its tasks, and sociodemographic questions. Respondents completed the survey by answering the questions in the same order. The survey could be saved and completed in multiple sessions. The researcher questionnaire was shorter than that for the general public.

The BWS section of both surveys was based on the same experimental design as described in online supplementary file 1. It consisted of eight tasks per respondent. In each task, the respondent was asked to select the most important, least important, second most important and second least important impacts from the list of eight impacts. The order of impacts in this list was randomised between tasks but was kept the same within a task.

#### Cognitive interviews

The questionnaire was tested in cognitive interviews with six members of the public and two researchers as detailed in online supplementary file 3. Cognitive interviews are a structured, systematic interview technique used to understand the cognitive processes respondents use when interpreting and responding to questions. The aim of the cognitive interviews was to test the near final survey instrument in terms of its wording and layout, and identify any aspects that might be considered ambiguous or cause confusion.^25^ The cognitive interviews proved valuable in refining the structure and wording of the survey instrument, in particular confirming that statements should be short, use varied wording to highlight differences between levels and be ordered randomly in each task, and that the maximum number of statements that it was manageable to consider in one task was eight. Further details are provided in online supplementary file 3.

#### Pilot

The revised survey instruments were then tested in a pilot with samples of MRC grant holders and the general public. This resulted in simplification of the BWS experiment to reduce respondent burden. Further details of the pilot are provided in online supplementary file 3.

**Figure 2:**
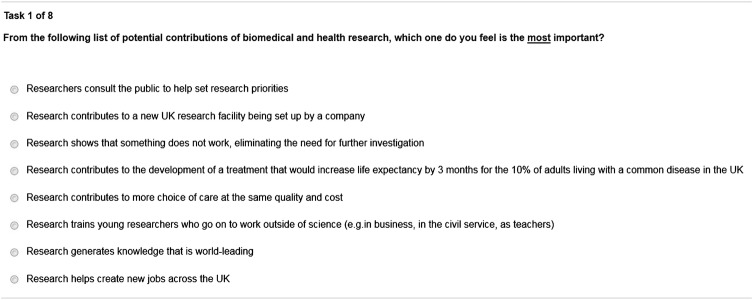
Example of best–worst scaling tasks.

Based on the results of the pilot and cognitive interviews, the attribute list was finalised in an internal workshop with all the researchers involved in the study. Final attributes and levels are presented in table 2 and an example task is presented in the screen shot in figure 2. Both questionnaires are provided in online supplementary file 4.

**Table 2:** Attributes and levels (domains and impacts) used in the surveys

	Level			
Domain	1	2	3	4
Knowledge (KNOW)	Research replicates the findings of others, helping to strengthen the evidence of how some things work (KNOW1).	Research results in a new finding, helping to focus subsequent research activities (KNOW2).	Research shows that something does not work, eliminating the need for further investigation (KNOW3).	Research reviews and combines previous findings, identifying areas of consistency and difference (KNOW4).
REF impact (IMPACT)	Research generates knowledge that is world leading (IMPACT1).	Research generates knowledge that is internationally excellent but which falls short of the highest standards of excellence (IMPACT2).	Research generates knowledge that is recognised internationally (IMPACT3).	Research generates knowledge that is recognised nationally (IMPACT4).
Training (TRAIN)	Research trains young people who go on to work in industry as scientists (TRAIN1).	Research trains young people, who become researchers and lecturers in universities (TRAIN2).	Research trains doctors and nurses who also become researchers (TRAIN3).	Research trains young researchers who go on to work outside of science (eg, in business, in the civil service, as teachers) (TRAIN4).
Jobs (JOBS)	Research helps create new jobs in the university (JOBS1).	Research helps create new jobs in one town (JOBS2).	Research helps create new jobs in one region (JOBS3).	Research helps create new jobs across the UK (JOBS4).
Private funding (PVT)	Research contributes to a follow-up study in the UK being funded by a company (PVT1).	Research contributes to an existing UK research facility being partly funded by a company (PVT2).	Research contributes to a new UK research facility being set up by a company (PVT3).	Research contributes to a company deciding to move a major part of its operations to the UK (PVT4).
Life expectancy (QOLY)	Research contributes to the development of a treatment that would increase life expectancy by 3 months for the 10% of adults living with a common disease in the UK (QOLYR, QOLYRC).	Research contributes to the development of a treatment that would increase life expectancy by 6 months for the 10% of adults living with a common disease in the UK (QOLYR, QOLYRC).	Research contributes to the development of a treatment that would increase life expectancy by 1 year for the 10% of adults living with a common disease in the UK (QOLYR, QOLYRC).	Research contributes to the development of a treatment that would increase life expectancy by 3 years for the 10% of adults living with a common disease in the UK (QOLYR, QOLYRC).
Cost of care (COST)	Research contributes to care being provided more cheaply without any change in quality (COST1).	Research contributes to better care being provided at the same cost (COST2).	Research contributes to better care being provided at a higher cost (COST3).	Research contributes to more choice of care at the same quality and cost (COST4).
Dissemination (DISS)	Researchers talk in schools about their research (DISS1).	Researchers give interviews to the media about their research (DISS2).	Researchers give public lectures about their research (DISS3).	Researchers consult the public to help set research priorities (DISS4).

REF, Research Excellence Framework.

10.1136/bmjopen-2015-010916.supp4 Supplementary file

### Survey implementation

The main stage of data collection was undertaken in February and March 2015. All 4620 researchers who did not participate or respond to the pilot were invited to take part in the main survey. In order to encourage researchers to participate, the study was publicised through the MRC's blog and Twitter, and individual researchers were sent up to three follow-up emails, each of which contained the link to the survey. A fresh sample of the general public was provided by ResearchNow, who hosted the survey, contacted respondents and collected the data.

### Data analysis

The data analysis in this study comprised two stages: (1) descriptive analysis and (2) modelling of the BWS data.

The aim of the descriptive analysis was to summarise the profiles of the participants in the general population and biomedical and health researcher samples by sociodemographic and other characteristics. We also conducted quality checks on the BWS data using three exclusion criteria as detailed in online supplementary file 5. The remaining data in both samples were then tested for representativeness against various sociodemographic characteristics (see online supplementary file 5 for further details). Finally, we constructed segments within the researcher and general population samples defined by research activity codes and their attitudes to science, respectively (see online supplementary file 5).

10.1136/bmjopen-2015-010916.supp5 Supplementary file

In the second stage of the analysis, modelling of the BWS data was conducted at the respondent level using discrete choice analysis.^26^ The aim of the modelling was to derive weights reflecting the relative importance of the research impact levels for different stakeholder groups. The probability of an individual respondent choosing a research impact level as the ‘most important’ (best) among a set of research impacts (attribute levels) can be modelled within a multinomial logit framework as described in online supplementary file 1. The estimated coefficients (weights) of each research impact can then be expressed on a common scale allowing one to infer how respondents or different groups of respondents value different types of research impact. In this stage, we also examined how individuals' preferences varied according to their attitudes to science (in the general population sample) and research activity codes (in the researcher sample).

## Results

### Data summary

Of the 4620 researchers invited, 1431 participated in the main survey questionnaire (response rate 31%). Out of the 1431 researchers, 465 provided partial responses to survey questions, including 260 researchers who did not complete any of the BWS tasks. As a result, the completion rate, defined as the proportion of researchers who provided no missing data (966) over the total number of participating researchers (1431), was 68% (table 3). The total number of researchers with fully or partially completed BWS tasks was 1171. Just over half (52.5%) of the respondents who completed the survey took more than 15 min to do so, while 12.5% completed it in 10 min or less.

**Table 3:** Response summary for both surveys

	Researchers	General population
Response rate	31%	NA
Number of total responses	1431	1113
Number of complete responses (no missing data)	966	1000
Number of respondents with missing data	465	113*
Number of respondents who completed at least one BWS scaling task	1171	1000
Survey completion rate	68%	90%
Observation exclusion criteria		
Completed 8 BWS tasks under 5 min	2 (0.2% of complete responses)	170 (2.2%)
Did not understand most of BWS tasks	37 (4% of complete responses)	102 (10%)
Unable to make comparisons in most BWS	146 (15% of complete responses)	129 (13%)

BWS, best–worst scaling; NA, not available.

We also received 1000 fully completed questionnaires from members of the general public who were members of the internet panel administered by ResearchNow. As is the norm with internet panels, we prespecified quotas to generate a sample representative of key characteristics including age, gender, social grade and geographic region. Given the nature of the sample, it is not possible to estimate a survey response rate. However, while none of the general public responses had missing data, ResearchNow estimated the completion rate to be 90%. The majority (67%) of the respondents who completed the survey took more than 15 min, while 7.6% completed it in 10 min or less.

Table 3 presents a summary of the quality checks on the BWS data (note: numbers are not exclusive to each category).

We checked the representativeness of the responses used in the modelling against various sociodemographic characteristics (see online supplementary file 5 for further detail). The general public respondents from the online panel were selected to be representative against the quotas set for gender, age and region, based on mid-2013 Office for National Statistics (ONS) population estimates. ResearchNow found it difficult to meet the quota for the Yorkshire and Humber region; hence, additional respondents were recruited from other northern regions instead. The distribution of 728 general public respondents who provided preferences for BWS choice models matched well with the targets for gender and region. Our sample under-represents the youngest age group and over-represents the oldest. It also contains a higher proportion of higher social grades than the general population.

The MRC provided age, gender and ethnicity of researchers in the grant database, and this was compared with the proportions observed in the survey responses used for modelling. Our sample contains a greater proportion of women than the overall population of MRC-funded researchers. It also over-represents white, mixed and black researchers, and under-represents Asian/Asian British researchers.

### Modelling

The preferences provided by the researcher and general public surveys were modelled separately, using the method established in prior BWS studies in other fields.^27–29^ The model results are presented in table 4. Each model coefficient in this table represents a preference weight measured as latent utility (which does not have a specific unit). To allow comparison between the preferences of researchers and the general public, we converted the coefficients of both groups to a common scale using a tangible unit—‘additional years of life expectancy’ (AYLE), based on each group's preferences for the life expectancy domain in the BWS task.

**Table 4:** General public model and researchers estimates

Model group		General public	Researchers
Description of research impact	Coefficient name	Coefficient† (95% CI)	Coefficient† (95% CI)
Research contributes to care being provided more cheaply without any change in quality.	COST1	4.582 (4.318 to 4.847)	4.087 (3.851 to 4.324)
Research contributes to better care being provided at the same cost.	COST2	4.613 (4.349 to 4.876)	4.632 (4.397 to 4.868)
Research contributes to better care being provided at a higher cost.	COST3	2.278 (2.029 to 2.527)	2.557 (2.322 to 2.793)
Research contributes to more choice of care at the same quality and cost.	COST4	4.349 (4.087 to 4.611)	3.168 (2.922 to 3.415)
Researchers talk in schools about their research.	DISS1	0.884 (0.668 to 1.1)	1.085 (0.866 to 1.304)
Researchers give interviews to the media about their research.	DISS2	0 NA	0 NA
Researchers give public lectures about their research.	DISS3	0.308 (0.097 to 0.519)	0.937 (0.719 to 1.155)
Researchers consult the public to help set research priorities.	DISS4	1.917 (1.677 to 2.157)	1.813 (1.576 to 2.05)
Research generates knowledge that is world leading.	IMPACT1	3.572 (3.305 to 3.839)	3.487 (3.248 to 3.726)
Research generates knowledge that is internationally excellent but which falls short of the highest standards of excellence.	IMPACT2	4.385 (4.12 to 4.649)	5.04 (4.804 to 5.277)
Research generates knowledge that is recognised internationally.	IMPACT3	3.817 (3.549 to 4.085)	3.868 (3.631 to 4.106)
Research generates knowledge that is recognised nationally.	IMPACT4	3.729 (3.466 to 3.992)	3.662 (3.424 to 3.899)
Research helps create a small number of new jobs in the university.	JOBS1	1.646 (1.419 to 1.874)	1.265 (1.048 to 1.482)
Research helps create a small number of new jobs in one town.	JOBS2	1.489 (1.263 to 1.715)	0.154* (−0.049 to 0.356)
Research helps create a substantial number of new jobs in one region.	JOBS3	1.832 (1.594 to 2.069)	1.153 (0.939 to 1.367)
Research helps create a substantial number of new jobs across the UK.	JOBS4	3.345 (3.081 to 3.61)	3.269 (3.036 to 3.503)
Research replicates the work of others, helping to strengthen the evidence of how some things work.	KNOW1	4.554 (4.287 to 4.822)	5.512 (5.274 to 5.75)
Research results in a new finding, helping to focus subsequent research activities.	KNOW2	2.618 (2.365 to 2.871)	3.418 ( 3.176 to 3.66)
Research shows that something does not work, eliminating the need for further investigation.	KNOW3	4.036 (3.766 to 4.305)	5.086 (4.847 to 5.325)
Research reviews and combines previous findings, identifying areas of consistency and difference.	KNOW4	3.521 (3.256 to 3.787)	2.876 (2.626 to 3.127)
Research contributes to a follow-up study in the UK being funded by a company.	PVT1	2.805 (2.553 to 3.056)	1.052 (0.839 to 1.265)
Research contributes to an existing UK research facility being partly funded by a company.	PVT2	2.794 (2.544 to 3.045)	1.07 (0.854 to 1.286)
Research contributes to a new UK research facility being set up by a company.	PVT3	2.796 (2.542 to 3.05)	1.699 (1.476 to 1.922)
Research contributes to a company deciding to move a major part of its operations to the UK.	PVT4	2.968 (2.707 to 3.229)	2.097 (1.859 to 2.335)
Research trains young researchers who become researchers in industry.	TRAIN1	3.713 (3.453 to 3.974)	3.081 (2.848 to 3.314)
Research trains young researchers who become university professors.	TRAIN2	3.368 (3.109 to 3.626)	3.768 (3.535 to 4.002)
Research trains young researchers who become doctors and nurses.	TRAIN3	3.775 (3.511 to 4.038)	2.832 (2.595 to 3.069)
Research trains young researchers who go on to work outside of science (eg, in business, in the civil service, as teachers).	TRAIN4	2.449 (2.193 to 2.704)	2.094 (1.857 to 2.332)
Value of change in 1 year on life expectancy of 10% of adults living with a common chronic disease in the UK	QOLYR	0.408 (0.363 to 0.453)	0.532 (0.493 to 0.57)
Intercept on life expectancy	QOLYRC	4.399 (4.139 to 4.658)	3.959 (3.727 to 4.191)
Impact statement position—bottom most	Bottom	0.128 (0.032 to 0.224)	0.395 (0.296 to 0.494)
Impact statement position—second from the top	Top2	0.145 (0.078 to 0.213)	NA
Impact statement position—top most	Top	0.192 (0.125 to 0.258)	0.171 (0.113 to 0.228)
Scale for second worst preference	Scale4	0.468 (0.437 to 0.499)	0.365 (0.342 to 0.388)
Scale for second best preference	Scale3	0.62 (0.583 to 0.656)	0.577 (0.551 to 0.602)
Scale for worst preference	Scale2	0.593 (0.557 to 0.629)	0.489 (0.462 to 0.517)
Scale for best preference(fixed to one)	Scale1	1 NA	1 NA

*p=0.132.

†p<0.05 for all estimated model coefficients except where explicitly specified.

NA, not available.

For example, the general public's preference for ‘research contributes to a company deciding to move a major part of its operations to the UK’ is equivalent to their preference for 7.21 AYLE for 10% of adults living with a common chronic disease in the UK (compared with 3.94 additional years for the researcher group). This conversion both facilitates the understanding of preferences in a tangible unit and allows comparison of preferences across groups and between populations. Further detail on the conversions is provided in online supplementary file 5.

**Figure 3:**
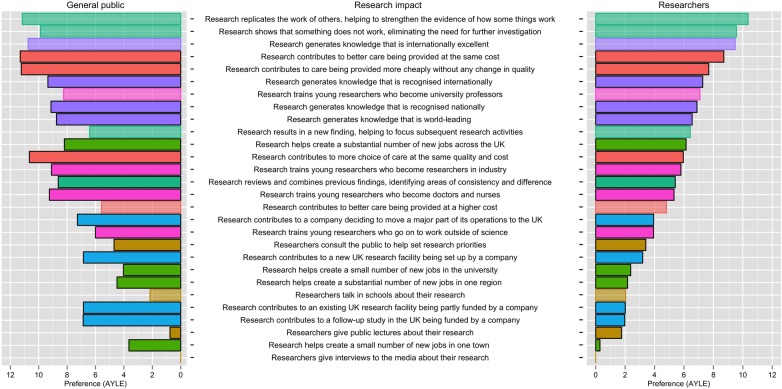
Preferences for different types of research impact, expressed as additional years of life expectancy (AYLE) for 10% of adults living with a common chronic disease in the UK. Note that the shaded (non-transparent) boxes illustrate impacts that are statistically different between the general public and researchers.

The preferences converted into equivalent values of life expectancy for the general public and researchers are presented in figure 3. The transparent bars indicate where there are not statistically significant differences between the two populations. Across the 28 different impacts there are statistically meaningful differences in 20 cases, suggesting that the general public and researchers value research impact in different ways. For example, the first horizontal bar in figure 3 relates to the impact statement ‘research replicates the work of others, helping to strengthen the evidence of how some things work’. This statement is valued at providing the equivalent of 11.16 (95% CI 9.77 to 12.55) AYLE by the general public and 10.36 (95% CI 10.12 to 10.60) AYLE by researchers. The transparent bars in the figure indicate that there is no statistically significant difference between the two groups with respect to this statement. By contrast, the fourth impact statement in figure 3—‘research contributes to better care being provided at the same cost’—is valued more by the general public, the solid bars indicating that this difference is statistically significant.

### Comparative analysis of preferences

Based on the analysis presented in table 4 and figure 3, we can infer a number of conclusions on how different types of research impacts are valued by the general public and researchers, summarised in box 2. The areas of agreement between the two groups are generally those relating to wider societal impact. For example, improved life expectancy, cost of healthcare and job creation (points 1–3) are all considered important by researchers and the public. However, the two groups differ in their relative valuation of training priorities, commercial capacity development and dissemination (points 6–8). We also find that both researchers and the general public rank research presented as ‘internationally excellent’ above that presented as ‘world leading’ (point 5). This is notable because the REF uses these terms in the opposite order when assessing academic quality.

Box 2 **Key observations arising from best–worst scaling analysis of the relative valuation of research impact**

1. Achieving higher life expectancy for adults living with a common chronic disease in the UK is one of the highest priorities for both the general public and researchers—well ahead of commercial and employment benefits.

2. Both researchers and the general public are concerned about the cost of healthcare provision, but the general public appears to be more cost-sensitive than the researchers.

3. Both researchers and the general public agree that creating a substantial number of jobs across the UK through research is important.

4. Public lectures, school talks and media interviews are among the least valued impacts by both the general public and biomedical and health researchers.

5. Research presented as internationally excellent is ranked higher than research presented as world leading by the general public and researchers, despite the Research Excellence Framework (REF) using these phrases in the opposite order.

6. The general public prefers the training of future medical professionals over the training of future academics, while researchers have the opposite preference. Overall, the general public gives much higher preference to the ‘training’ domain of impacts compared with the researchers.

7. The general public makes no distinction between different types of commercial capacity development. Researchers are more nuanced showing a preference for attracting foreign investment. The general public also attaches a much higher preference to this domain compared with the researchers.

8. In the ‘dissemination’ domain the general population values all research impacts higher than researchers, except the impact ‘researchers give public lectures about their research’ which is valued more by the researchers than the general public.

For the two group-level models, we also tested differences in preferences between segments of both populations. For researchers, the segments were based on research activity codes.^24^ For the general public, each respondent was assigned to a segment based on their attitudes towards science, as defined by the set of questions extracted from the Public Attitudes to Science 2014 study.^23^ Details of how we implemented this segmentation and the results are provided in online supplementary file 5. Overall there were only minor differences between the general public segments, and when they occurred they were difficult to coherently interpret. For the researchers, the differences by HRCS code were more pronounced and had a degree of face validity. For example, ‘health services researchers’ were more concerned about healthcare costs than those involved in ‘underpinning research’.

## Discussion

We can identify three key findings from this study. First, that it is possible for different types of impacts to be directly compared and rated, and that BWS offers a potentially effective way to make such comparisons. Second, that there are similarities in views between the researchers and the public about the relative importance of social impacts, but also notable differences of opinion between these groups regarding other research-related impacts. These differences are important, as researchers are increasingly asked to make judgements about the value, or potential value, of research in the award of public funding. Finally, we note that our findings differ from those of a previous study by Miller *et al*,^16^ suggesting that further research is required. We explore each of these in turn.

The study shows the potential of BWS as a methodology for the quantitative assessment of research impact. While a stated preference approach has been used in a recent Canadian study,^16^ to the best of our knowledge, this is the first application of the BWS approach for relative valuation of research impacts. The BWS methodology allows quantification of preferences and we have also demonstrated how these preferences can be compared across two discrete groups on a common scale. Compared with stated preference discrete choice experiments, the BWS methodology uses a simpler choice task involving less cognitive burden for respondents and can accommodate a larger number of attributes within each choice task. Another strength of the study is that it uses large, national samples of MRC-funded researchers and the general public. To the best of our knowledge, this is the first study to contrast valuation of research impact between UK biomedical researchers and citizens. Within a given sample, BWS enables comparison of valuations of research impact by simply comparing the marginal utility estimates; this is an improvement over discrete choice experiments, which unless carefully designed, do not allow for comparisons of attribute levels (research impacts) across different attributes (domains).^30^

The second principle finding is that the general public and researchers value research impacts in different ways. However, it is also the case that when the two groups are in agreement, this is generally about matters that are seemingly more important and associated with wider social benefit (eg, life expectancy, cost of healthcare, job creation), rather than impacts associated with the research system, where the two groups tend to disagree (eg, training, commercial capacity development). Either way, this is important as the UK research councils ‘encourage researchers to consider the potential contribution that their research can make to the economy and society from the outset, and the resources required to carry out appropriate and project-specific knowledge exchange impact activities’.^31^ As part of their funding applications, researchers must submit a ‘pathway to impact’ statement which is peer reviewed by referees and panel members. Similarly, the funding councils assessed research impact using a case study approach as part of the 2014 REF. These case studies were reviewed by academic peers and non-academic experts providing a private, public and third sector perspective. However, in assessing the value of the impact claimed, reviewers cannot currently draw on comprehensive evidence of the views of beneficiaries (ie, the general public) or the producers of research (ie, biomedical and health researchers) to qualify or justify their grading. Indeed an evaluation of how panels assessed research impact as part of REF 2014 highlighted this as a concern raised by panel members.^32^ In other words, the subjective valuation of research impacts rests on weak empiric foundations. This in turn raises questions about the reliability of impact assessment and whether current processes are robust, fair and transparent. The research presented in this paper is a small first step in understanding how research impacts can be valued. With further research, it may be possible to use such valuations to develop metrics for assessing research impact, although we stress that this is a longer term objective, to be considered alongside the need for better ways of identifying and measuring societal impact more generally, and should not be advocated based on the current study.

Our final key finding is that the results observed differ from a previous study in this area. To the best of our knowledge, this is only the second time that a discrete choice experiment has been used to assess how the general public and researchers value different types of research impact, and the first time that BWS has been used to elicit these choices. The results of the current study are different to those of Miller *et al*^16^ who argued that the similarities between the general public and researchers were more important than the differences. We do not believe that the difference between the two studies can be explained on methodological grounds (ie, the stated preference method vis-à-vis BWS). That said, there were two important differences between the studies. First, the choice context is different. Miller *et al* asked respondents to review and assess the impacts using a scenario of an academic biomedical research team. Behaviourally, this may induce respondents to respond as if wearing the hat of an ‘expert reviewer’ rather than as lay public. The same applies for researchers. Here, we presented research impact in much broader terms and not within a given scenario. Second, the selection of attributes in Miller *et al* remains within the strict confines of academic-oriented contributions, namely publications, trainees, patents and targeted economic priorities. In this study, we attempted to elicit valuations from a much broader range of domains and research impacts within each domain, perhaps also reflecting the Research Council UK's (RCUK) definition of research impact where ‘academic impact forms part of the critical pathway to economic impacts and societal impact’. That said, it may also be that there are cultural differences between the Canadian and British respondents or other reasons for the differing results. In any case, given the importance of research impact assessment, it seems that these two studies need further refinement and replication both in Canada and the UK, but also in other countries and for other disciplines.

We note four areas that would merit particular focus in further studies. First is the need to improve engagement of researchers. A large proportion of respondents in the researcher sample dropped out prior to fully completing the survey (32%). A review of the qualitative feedback submitted through the survey questionnaire suggested that researchers either felt the survey was politically motivated; that it did not cover all important aspects of research impact; or they wished that a ‘none’ option was available in the BWS tasks. Second, a limitation of this study is the representativeness of the internet recruited panel of the general public. While we were able to match key demographics such as age and region of residence with mid-2013 ONS population estimates, the significantly higher proportion of individuals in the higher National Readership Survey (NRS) Social Grades in the sample provides an indication that the profile of respondents related to other characteristics (eg, education) may be significantly different and thus not representative of the general population in the UK. Third, although we were able to detect differences in viewpoints between members of the public and researchers, we were not able through this study to understand the reasons for these differences between groups. Encouraging discussions around why preferences differ in specific instances might usefully inform future research objectives, as well as encourage more nuanced communication between researchers and the public on the potential benefits of research. Finally, further work in this area could start to work towards the longer term goal of being able to rate and compare different types of impact to support decision-making.

To summarise, this work sets out a new approach to elicit opinions about the relative importance of different types of research impact and highlights evidence for some important differences in opinion between researchers and members of the public. This has implications for policy-making, since researchers and funders commonly assess the potential and realised impact of research as part of funding decision-making processes. The methods set out here might offer one way to understand and begin to address this, with the potential, through further research, to develop a way to assess and compare different types of impact based on empirical evidence of their relative importance to members of the public. Exploring this question and these methods further could help better align publicly funded research with the needs and priorities of the public, strengthening accountability and public engagement with science, and perhaps, ultimately, offering better value to society.
