# Supplementary material for: Understanding the relative valuation of research impact: a best–worst scaling experiment of the general public and biomedical and health researchers
Source: BMJ Open. 2016 Aug 18;6(8):e010916. doi: 10.1136/bmjopen-2015-010916 (PMC5013498; doi:10.1136/bmjopen-2015-010916)
Supplement: Supplementary file [file bmjopen-2015-010916supp1.pdf]

Supplementary File 1: Best Worst Scaling method

Design of the Best Worst Scaling Experiment

The tasks in the BWS experiment were specified using an orthogonal main-effects experimental design that allowed 8 research-impact domains with 4 levels of impact to be tested. An orthogonal design allows estimation of the main effects of all research-impact levels independently and has the desirable property of level balance, which ensures that all levels of all the research-impact domains are equally used in the design (thus they have same likelihood of being presented to a respondent). The generated experimental design matrix included 32 rows with each row corresponding to a possible combination of research-impact levels, which represents a BWS task. As it might have been cognitively difficult for one respondent to evaluate all 32 BWS tasks, the design was divided into four blocks so that each respondent was presented with eight BWS tasks. Blocking was specified such that there was no correlation between blocking and research-impact levels. The coding of research-impact levels was chosen to avoid designing tasks (where applicable) showing every research-impact at its 'best' level; with every research-impact level at its 'worst' level, and ‘easy to choose’ tasks.

Modelling Framework

The aim of the analysis is to derive weights reflecting the relative importance of the research-impact levels for the general public and biomedical and health researchers on a common scale. Assuming a Random Utility Maximisation framework (McFadden, 1974) and that the random components of the utilities are Extreme Value type 1 (EV1) distributed enables the choice data to be analysed using the conditional multinomial logit (MNL) model (McFadden, 1974; Train, 2003). Thus the choice of 'best research-impact' (i.e., most important) given a set of research impact levels can be described within an MNL as follows (Flynn and Marley, 2014):

$$P_i(Best\ Research - Impact|c) \ = \frac{exp(V_i)}{\sum_{j \in c} V_j}$$

Where:

P_i is the probability of selecting the 'most important' (best) research-impact level *i;*

c denotes the subset of choice alternatives – i.e., research-impact levels that appear in a given BWS task out of a total *C* choice alternatives in the experimental design of the study;

V_i is the observed utility for research impact level *i equal to* β_i ** Research-Impact*$_i$.

The corresponding MNL model for the choice of the 'least important' (worst) research impact can be written as (Marley and Louviere, 2005):

$$P_i(Worst\ Research - Impact|c) \ = \frac{exp(-V_i)}{\sum_{j \in c}(-V_j)}$$

where $-V_i$ is the utility of the 'least important' research impact.

We can similarly write the probability for 'second most-important' and 'second least-important' research impact. As per Hensher and Bradley (1993), the variation in the quality and nature of

responses across 'most important, 'least important', 'second most-important and 'second least-important' is captured through a corresponding set of scale parameters. As shown in Figure SF1.1, Subtask 1 corresponds to the subset of BWS data in which respondents provided their choice for the 'most important' research impact and Subtask 2 corresponds to the subset of BWS data for choices of the 'least important' aspect of research impact. Subtasks 3 and 4 correspond to the subsets of BWS data for the 'second most important' and 'second least important' research impacts, respectively. For model identification purposes, the scale of Subtask 1 data (σ_1) is set equal to one so that the scales of the other three subsets of data (σ_2, σ_3 and σ_4) are estimated relative to the scale of Subtask 1 data.

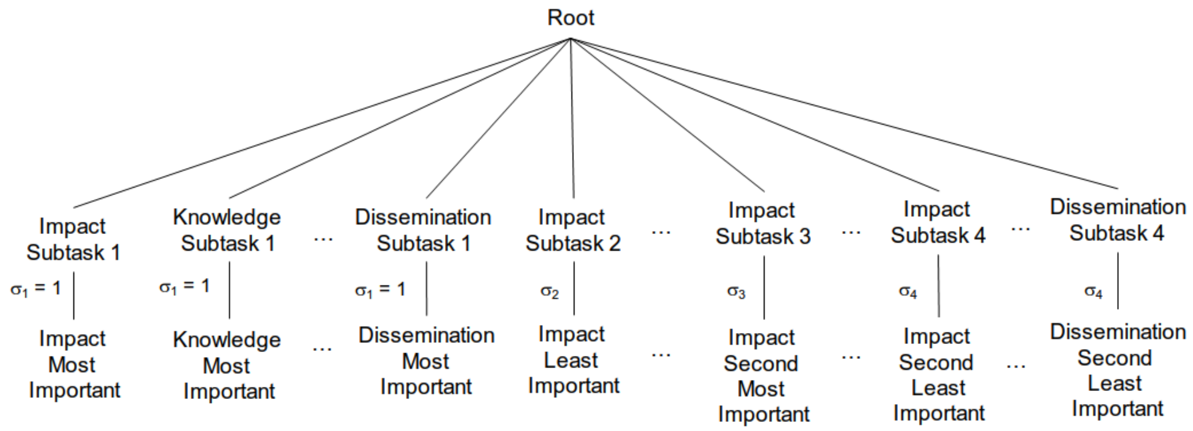

Figure SF1.1. BWS model estimation structure

All parameters are estimated relative to a single research-impact level, which is assigned the value of zero. Hence, estimates represent a complete ranking of research-impact levels on a common scale and relative to that research-impact level having zero weight. The analysis of the BWS data is also aimed at capturing potential bias related to the positioning of the research impacts in the BWS task – that is, whether respondents are more likely to select research impacts that appear at the top or bottom of a BWS task. We therefore specify and estimate the effect of dummy variables indicating the position of the selected research-impact (i.e., top, bottom).

The analysis reported in Supplementary File 5 explores the variation of preferences across different segments of the general-population and researcher samples. For example, do some groups of researchers prioritise certain dimensions of impact differently from others; do some groups in the general population rate particular research impacts higher than others? Also in Supplementary File 5, we contrast the valuations of research impacts between the biomedical researchers and general public. Given that the BWS parameter estimates between researchers and the general population are directly comparable, we express valuations in Years of Life Expectancy.

References

Flynn TN, Marley AAJ. Best-worst scaling: theory and methods. In: Hess S, Daly A, eds. *Handbook of Choice Modelling,* Cheltenham, UK: Edward Elgar Publishing 2014: 178-201.
Hensher D, Bradley M. Using stated response choice data to enrich revealed preference discrete choice models. *Marketing Letters* 1993; 4(2), 139-151.

Marley AAJ, Louviere JJ. Some probabilistic models of best, worst, and best-worst choices. *Journal of Mathematical Psychology* 2005; 49: 464-480.
McFadden D. Conditional logit analysis of qualitative choice behaviour. In: Zerembka P, ed. Frontiers in Econometrics, New York: Academic Press 1974, 105-142.
Train K. Discrete Choice with Simulations. Cambridge, UK: Cambridge University Press, Cambridge 2003.
