# Supplementary material for: Understanding the relative valuation of research impact: a best–worst scaling experiment of the general public and biomedical and health researchers
Source: BMJ Open. 2016 Aug 18;6(8):e010916. doi: 10.1136/bmjopen-2015-010916 (PMC5013498; doi:10.1136/bmjopen-2015-010916)
Supplement: Supplementary file [file bmjopen-2015-010916supp2.pdf]

Supplementary File 2: Defining and categorising the impacts of biomedical and health research

Literature review

Methods

The aim of the literature review was to:

- Identify a wide range of potential impacts of research
- Investigate different ways of classifying impacts
- Produce a possible long list of types of impact and domains for impact that could be tested in focus groups and interviews

We wanted to understand what classification approaches existed and what types of impact would be likely to resonate with both the groups we intended to survey: academics and the general public. There are a number of well-known frameworks and approaches to investigate research impact in the academic sphere, so in that area we focused on conducting a review of known academic approaches to classifying research impacts and the measures of research impact used. To this, we added a review of public perceptions of research impact. It was less clear what information would be available in this area, so as well as capturing any classification systems or metrics and measures of research impact, we also aimed to identify general lessons about the way in which our survey should be structured and questions worded for comprehension and clarity.

The review of existing frameworks and approaches largely covered grey literature as well as some academic literature and focused initially on a limited set of key sources known to the project team and advisory board. The review of academic funding and evaluation schemes focused on the UK but drew on international examples. The following research evaluation frameworks and systems were investigated:

- Snowball Metrics
- Canadian Academy of Health Sciences (CAHS)
- Excellence in Research for Australia (ERA)
- National Institute for Health Research (NIHR) dashboard
- Research Excellence Framework (REF)
- SIAMPI (**S**ocial **I**mpact **A**ssessment **M**ethods for research and funding instruments through the study of **P**roductive **I**nteractions between science and society)
- Agence d'évaluation de la recherche et de l'enseignement supérieur (AERES)
- Research Councils UK Outcomes System (ROS)
- Payback Framework
- National Institutes of Health (NIH)
- National Science Foundation (NSF)
- Consortia Advancing Standards in Research Administration Information (CASRAI)
- Researchfish

From each framework, we gathered the following information using a simple excel spreadsheet:

- Description of source
- Web link or reference
- Summary of the framework and its aims
- Overview of the categorisation approach used in the framework
- List of titles of the categories used
- List or summary of the indicators falling into each category

We then mapped the categories for each framework against an 'expanded' Payback Framework comprising the following: knowledge production; research targeting; capacity building; informing policy; product development; health benefit; health sector benefit; economic benefit; social benefit. The mapping was based not just on the titles of the categories in each framework, but also the indicators contained within them. Each category could fall within more than one section of the Payback Framework. Based on this mapping, we started to develop a possible classification system for types of impact, aiming to avoid overlap between categories. We mapped the indicators from each framework against this classification system and summarised the types of indicators, and hence impacts, falling into each domain.

The review of public engagement and perceptions literature focused on UK science communication and funding bodies, with some additional searching of the academic literature from Google Scholar. A formal search and review approach was not used. The following sources were used:

- Sciencewise
- Science Media Centre
- Wellcome Trust
- James Lind Alliance
- National Coordinating Centre for Public Engagement
- Medical Research Council
- Google Scholar

Findings

Our light touch review of the public engagement literature in this area suggests that most of the literature currently available is only peripherally relevant to this study. Much of the work is focused broadly on how research can involve the public, and work on research impact has tended to focus on setting research priorities in particular research areas through consultation with patients and carers. As such, we were not able to identify any clear metrics or frameworks that we can use as a basis for our survey development. However, we were able to identify several interesting examples and general lessons which were valuable in developing the structure and wording of the survey.

In terms of the public's understanding of research, there is some evidence that confusion between research and healthcare can arise, with, for example, patients and carers focusing more on care and recovery needs than research questions in a research priority setting exercise (Owens et al., 2008). Nevertheless, despite the suggestion that the public will favour research with a clear application over basic discovery research, the UK's 2014 *Public Attitudes to Science* survey shows that 78% of adults agree with the statement "Even if it brings no immediate benefits, scientific research which advances knowledge should be funded by the Government" (Castell et al., 2014). This is consistent with Miller's (2004) observation in the US of substantial support for basic research among the general population: in the National Science Board's *Science and Engineering Indicators* survey the

proportion of respondents agreeing with that survey's equivalent statement had remained at approximately 80% over the 15 years prior to 2000.

Evans and Durant (1995) demonstrated that public support for research varies by the area of science and in relation to a number of other factors. In particular, they looked at the relationship between knowledge and attitudes. While they found some evidence that higher levels of scientific knowledge are associated with more supportive attitudes towards science, both in general and for 'useful science', they also observed that the well informed were more strongly opposed to morally contentious or 'non-useful' areas of research. Similarly, level of interest in a topic has been found to correlate with the perceived benefits of research in that area (OST/Wellcome Trust, 2000). These observations raised questions around whether the questions in the BWS element of the survey statements should be worded generically or relate to specific areas of science.

This work also highlighted a tendency to agree with statements, particularly among those less well informed about science (Evans & Durant, 1995). This was noted as a potential issue for our focus groups, and was important in considering whether statements should be framed positively or negatively in the survey.

In our analysis of academic frameworks, we identified three main classification approaches:

- Academic focused frameworks, which concentrated on measures around volume and quality of outputs, esteem of researchers, and capacity building. Sometimes they also included one general category for wider impacts. Examples here include ERA, ROS, AERES, NIH.
- Frameworks which were focused on wider, non-academic impacts (e.g. REF, NSF)
- Logic model based approaches – Snowball Metrics, NIHR dashboard , Payback Framework and CAHS

A summary of the types of impacts captured by each of the frameworks investigated is provided in Figure SF2.1 below.

Figure SF2.1 Frameworks and the impacts they capture

Source	Groupings
Snowball Metrics	Input/process/output is primary classification. Also uses Research grants/Postgraduate education/Enterprise activities
CAHS	• Advancing knowledge<br>• Capacity building<br>• Informing policies and product development<br>• Health and health sector benefits<br>• Broader socio-economic benefits.<br>Advancing knowledge includes new discoveries and breakthroughs from health research and contributions to scientific literature<br>Capacity building includes development and enhancement of research skills for individuals and teams.<br>Informing decisionmaking includes the impacts of research in areas of science, public, clinical and managerial decisionmaking practice policy<br>Health impacts include advances in prevention, diagnosis, treatment and palliation when related to research
ERA	Indicators of: research quality, research volume and activity, research application, recognition
NIHR dashboard	Areas are financial, internal, external. But pilot dashboard seems to be goal focused rather than based on these areas.
REF	Economic impacts<br>Commercial impacts<br>Impacts on public policy and services<br>Impacts on society, culture and creativity<br>Health and welfare impacts<br>Production impacts<br>Impacts on practitioners and services<br>Impacts on the environment<br>Impacts on international development
SIAMPI	Direct interaction, indirect interaction, financial interaction
AERES	• Productivity (quality, quantity, impact of scientific research)<br>• Socio-cultural and economic impact (national and international relevance of the research)<br>• Strategy and research life (management, development of research teams and so on)<br>• The 'project' performance (originality, quality, prospects and so on).
ROS	Publications (including journals, books and conferences)<br>Other Research Outputs (including biological, creative, electronic and physical)<br>Collaborations and Partnerships<br>Dissemination and Communication<br>Intellectual Property and Exploitation<br>Awards & Appointments<br>Staff Development<br>Further Funding<br>Impact
Payback Framework	Knowledge production, research targetting and capacity building, informing policy and product development, health and health sector benefits, economic and societal benefits
NIH	Significance, investigator, innovation, approach, environment
NSF	NSF values the advancement of scientific knowledge and activities that contribute to the achievement of societally relevant outcomes. Such outcomes include, but are not limited to: full participation of women, persons with disabilities, and underrepresented minorities in science, technology, engineering, and mathematics (STEM); improved STEM education and educator development at any level; increased public scientific literacy and public engagement with science and technology; improved well-being of individuals in society; development of a diverse, globally competitive STEM workforce; increased partnerships between academia, industry, and others; improved national security; increased economic competitiveness of the United States; and enhanced infrastructure for research and education.
CASRAI	Research impact on capacity, research impact on productivity, research impact on society
Researchfish	Publications, collaborations, fundings, career destinations, skills, dissemination, policy impacts, materials, IP, products, spin outs, recognition, facilities, other.

Mapping the categories from the different frameworks against the expanded Payback Framework, we make a number of observations. Firstly, we noted that knowledge production and research targeting are closely related, with both being based on the direct academic outputs of research, with some categories sitting within both columns. We also observe significant overlap between product development and economic benefit, with categories such as 'enterprise activity' in Snowball Metrics, and 'commercial impacts' and 'production impacts' from the REF sitting across them. In many frameworks, policy development is grouped with public services. This is because in most cases a wider view of policy is taken, rather than just focusing on guidelines which are often grouped instead as health improvements, and indeed this approach may be easier to understand for a general audience. This may also reflect the fact that a number of these frameworks are intended to assess a wider portfolio of research, not just health research. The Payback approach also does not capture indicators around dissemination and communication of research to the general public through outreach activities and media engagement. A summary of the mapping of the frameworks against the expanded payback framework is shown in Figure SF2.2 below.

Figure SF2.2 Mapping of frameworks against the expanded payback framework

Framework	Knowledge production	Research targeting	Capacity building	Improving policy	Product development	Health benefits	Health sector benefits	Economic benefit	Social/societal benefit	Other
Snowball Metrics		Research grants	PG education		Enterprise activity			Enterprise activity		
ERA	Research quality; research volume and activity		Recognition		Research application					
REF				Impacts on public policy and services	Commercial impact, production impact	Health and welfare impact	Impacts on practitioners and services	Economic impacts, commercial impact, production impact	Impacts on society, culture and creativity, impacts on the environment, impacts on international development	
AERES	Productivity, the 'project' performance		Strategy and research life					Socio-cultural and economic impact	Socio-cultural and economic impact	
ROS	Publications, other research outputs	Other research outputs, further funding	Collaborations and partnerships, awards and appointments, staff development		IP and exploitation	Impact	Impact	Impact	Impact	Dissemination and communication
NSF			Full participation of women, persons with disabilities, and underrepresented minorities in science, technology, engineering, and mathematics (STEM); enhanced infrastructure for research and education; improved STEM education and educator development at any level	Increased partnerships between academia, industry, and others	Increased partnerships between academia, industry, and others			Development of a diverse, globally competitive STEM workforce; increased economic competitiveness of the United States;	Improved STEM education and educator development at any level; increased public scientific literacy and public engagement with science and technology; improved national security; improved well-being of individuals in society;	
CASRAI	Impact on productivity: quantity of direct knowledge and innnovation production; quality of direct or indirect knowledge and innovation production; quantity of derived knowledge and innovation production.		Impact on capacity: human capacity; leadership capacity; physical capacity; integration capacity.		Impact on productivity: quantity of direct knowledge and innnovation production; quality of direct or indirect knowledge and innovation production; quantity of derived knowledge and innovation production.	Impact on society: Direct economic benefits to society; indirect economic benefits to society; broader social benefits; broader health benefits; broader environmental benefits; broader cultural benefits.	Impact on society: Direct economic benefits to society; indirect economic benefits to society; broader social benefits; broader health benefits; broader environmental benefits; broader cultural benefits.	Impact on society: Direct economic benefits to society; indirect economic benefits to society; broader social benefits; broader health benefits; broader environmental benefits; broader cultural benefits.	Impact on society: Direct economic benefits to society; indirect economic benefits to society; broader social benefits; broader health benefits; broader environmental benefits; broader cultural benefits.	
Researchfish	Publications	Funding	Collaborations, career destinations, skills, materials, recognition, facilities	Policy impacts	IP, products			Spin outs		Dissemination

Based on these observations, we developed a possible categorisation approach with the aim of minimising the overlap between the different domains. The domains we identified were as follows:

- Knowledge production and research targeting (KPRT)
- Capacity building (including training, network building, awards and other esteem measures, widening of participation in research, infrastructure) (CB)
- Innovative and economic impact (IEI)
- Health and health sector benefit (HHSB)
- Policy and public services impact (e.g. environment, education (outside of academia), safety and security) (PPS)
- Public engagement, dissemination, culture and creativity (PEDCC)

To investigate this further and to serve as a basis for the types of impacts to be investigated in the survey, we mapped the indicators from each of the frameworks against these domains to understand what level of overlap remained between the domains and what type of indicators

are already used to capture impacts in these areas. We found that the indicators contained within each domain were as follows:

- KPRT: Volume and quality measures of knowledge outputs. Future funding was also included here.
- CB: Esteem measures; number and quality of researchers trained; collaboration and networking (academic and wider); wider participation in research.
- IEI: New products and processes developed (and patented, licensed, used); new businesses (spinouts); benefits to companies in terms of profitability, new clients, competitive advantage, efficiency etc.; job creation, workforce development and increased economic competitiveness of region/ country.
- HHSB: Impact on guidelines/policy/professional training or development in health; impact on practice including saving NHS money, making processes more efficient or resilient etc.; impact on health and well-being (of a group of patients, or more widely).
- PPS: Changes to policy outside of health; improvements in the delivery of public services (outside healthcare) including improved education, national security, international development, environment (including more efficient services, cost savings etc.); benefits to public wellbeing and society more widely resulting from these changes.
- PEDCC: Number and range of dissemination and outreach activities; increased public understanding of and engagement with science.

Broadly, the overlap between categories is more limited using this framework and typically only seems to occur when the indicators are very broad. For example, there may be some overlap between health and public policy when looking at frameworks which are not focused on health specifically and hence do not have health specific indicators. We also note that there are a limited number of indicators identified in the public engagement/dissemination category. A summary of the types of indicators in each domain is provided in Figure SF2.3 below. It is interesting to note that in several of these domains there is some 'progression' in terms of the level of impact between the types of impact generated. For example, in the health and health sector benefit domain, an impact on guidelines, policy or training can be translated into improvements in practice, including efficiency and cost savings, which can ultimately result in improved health and wellbeing for either a group of patients or a wider population.

Figure SF2.3: Draft categorisation of impacts, developed from the existing literature

Category	Knowledge production and research targeting	Capacity building	Innovative and economic impact	Health and health sector benefit	Policy and public services (other than health)	Public engagement, dissemination, culture and creativity
Types of impacts and measures of those impacts included in the category	Volume and (qualitative and quantitative) quality measures<br><br>Future funding	Esteem measures<br><br>Number and quality of researchers trained<br><br>Collaboration and networking<br><br>Wider participation in research	New products and process developed<br><br>New businesses (spinouts)<br><br>Benefits to companies<br><br>Job creation, workforce development and increased economic competitiveness	Impact on guidelines /policy /professional training or development in health<br><br>Impact on practice including saving NHS money<br><br>Impact on health and well being	Changes to policy outside of health<br><br>Improvements in the delivery of public services (outside of health)<br><br>Benefits to public wellbeing and society more widely resulting	Number and range of dissemination and outreach activities<br><br>Increased public understanding of and engagement with science

Focus groups

Focus groups with members of the public and interviews with individual researchers were used to refine the impact statements to be used in the survey. This preliminary research aimed to identify: (1) the impacts that the public and researchers expect to come from health and biomedical research; (2) whether there is a common understanding of these impacts; (3) how these impacts should be categorised; and (4) how these impacts might be measured.

Four focus groups were held with the general public, conducted in two waves. Between the waves the topic guide was fundamentally reviewed and revised to ensure that this stage of the research learned as much as possible about public understandings.

The sample for the first wave of focus groups comprised:

- group 1: two women and three men aged 24-45 from social grades ABC1; and
- group 2: five men and five women aged 46-70 and from social grades C2DE.

The groups took place in April 2014 in Hitchin, a small town just north of London, in the commuter belt. Both groups lasted 90 minutes and were audio recorded. The topic guide can be found below.

The sample for the second wave of focus groups comprised:

- group 3: five women and five men aged 25-45 from social grades C2DE; and
- group 4: five men and five women aged 46-70 from social grades ABC1.

The groups took place in May 2014 in Leeds, a large city about 200 miles north of London. Both groups lasted 90 minutes and were audio recorded. Again, the topic guide can be found below. A major change from wave 1 was the division of the groups into two subgroups for the second half of the session, which encouraged input from all participants on a greater number of potential impacts.

Recruitment was contracted to Plus4 Market Research, using the recruitment questionnaire included below. People with a background in the following areas were excluded from the discussions: market research, journalism, public relations, marketing, scientific research, doctor, nurse, dentist, optician, alternative medicine. The recruitment process also excluded those who had attended focus groups on any subject in the last six months and anyone who had attended a focus group on health or research in the last two years.

In addition to some more detailed observations on the wording of individual statements, the main findings from the focus groups were as follows:

Definition of research

Following brainstorming on the evocation of the word ‘research’ participants agreed with our proposed definition that research is “studying something so that we (as humankind) can understand better how it works”.

Health research and medical research were seen as slightly different, with health research considered as a broader term relating to research into health and lifestyle, understanding causes, and understanding ‘who suffers from what’. In contrast, medical research was considered as being more technical, focused on looking for cures and usually thought of as concerning drug development.

Research impact

According to participants, research impacts from health and medical research were focused on better health, better quality of life and longevity. Hence they generally saw the purpose of medical research as producing cures and ways of preventing illness and, to a lesser extent, improving palliative care.

Biomedical and health research process

Generally, little was known about research processes, infrastructure and practices such as academic journal publications. As noted above, perceptions of medical research were focused on drug development, with a focus on testing.

Trust

Participants tended to trust academic researchers more than those working in commercial environments, as they felt they were motivated by a desire to do good and an interest in their subject, rather than by a desire to make money.

Wave 1 Topic Guide

Introduction	(2 mins)
	Introduce self
	Introduce anyone else who is observing or helping
	Has anyone been to anything like this before?
	I have here a list of things I'd like to cover but really want to hear your views on the issues we'll be introducing.
	There are no right or wrong answers. Everyone is entitled to their own view, so I'd like to hear from everyone because everyone's view is valid.
	You don't have to answer all of the questions.
	You are free to leave before the end of the session, if you wish.
	I would like to tape record the discussions, just to save me taking notes, so I can listen to what you're all saying.
	No one will be identified in the report. All the information will be collected together and anonymised.
	This is just one session of 4 that we are running around the country on this project.
	Is everyone happy for me to record the session?
	Please could everyone turn off their mobile phone.
	SWITCH ON RECORDER
	I will tell you more about the project and the client at the end of the session.

Round robin	(5 mins)
OK, let's go round and introduce everyone. Please just give your first name, if you work what you do, who you live with and how many children you have, if any, and lastly – what do you understand by the term 'research'?<br><br>IF QUESTIONED ABOUT THE TYPE OF RESEARCH – EXPLORE WHY THAT MATTERS BUT TRY TO KEEP VERY TOP LEVEL FOR EXAMPLE: Do the different types of research have anything in common?<br><br>INTERVIEWER TO LIST RESEARCH UNDERSTANDINGS ON POSTCARDS	
Defining research	(10 – 15 mins)
PLACE ALL POSTCARDS ON TABLE	
ASK PARTICIPANTS TO:<br>• expand on each and annotate the cards as necessary.<br>• group the cards and then explore why they have grouped them as they have<br>• pick out the cards relevant to health and biomedical research and explore why these chosen and others not	
TRY TO SUM UP DIFFERENCES AND SIMILARITIES AND GET PARTICIPANTS TO AGREE THAT RESEARCH IS SOMETHING ALONG THE LINES OF:	
**Studying something so that we (as humankind) can understand better how it works.**<br><br>**Biology: understanding how bodies work, physical sciences: understanding how the natural world works, social science: understanding how societies work, humanities: understanding how cultures work.**<br><br>How would this definition apply to health and biomedical research?	
Do you think that there is any difference is between health research and biomedical research? If so, how would you define each?	
Probe for health and biomedical research examples.	
Do you want to change how you have grouped your definitions of research or to add anything?	
Research outcomes	(50 mins)
What sort of things would you expect to come out of health and biomedical research?<br><br>EACH PARTICIPANT TO FILL IN POSTCARDS<br><br>FOR EACH EXPLORE:<br>• What is it (define/explain as necessary) and why it has been included?<br>• Who would benefit/suffer as a result of that outcome?<br>• Are there other types of people who would benefit or suffer as a result of health and/or biomedical research? PROBE IF NECESSARY TO STIMULATE IDEAS: Patients, researchers, the public, children, elderly people, carers, doctors, nurses, private companies, general public.<br>• What for you would be the biggest impact of health and biomedical research?	
ONCE RUN OUT OF IDEAS INTRODUCE ANY FROM THE LIST BELOW THAT HAVE NOT EMERGED<br><br>What about:<br>1. New knowledge and ideas, perhaps leading to further research<br>2. New products registered	

3. Changes to Government policies or new Government policies
4. Changes in healthcare/diagnosis/treatment methods
5. Changes in health services e.g. the way healthcare is delivered by GPs rather than hospitals
6. Changes in costs
7. Publications – where published/how many published
8. Awards/professional progression/peer recognition for researchers
9. Employment
10. Training
11. National security
12. Science education for students
13. Changes to the environment
14. Funding for research
15. Economic climate
16. Public safety

IF MORE THAN 15 IN TOTAL, WORK TO REDUCE THE NUMBER.

THIS CAN BE DONE AS PART OF EXPLORING WHAT EACH MEANS

OK TO LEAD A BIT HERE IF NECESSARY

Are some of these really the same thing expressed differently?

Are some of these the same idea but a difference of scale or quality?

FOR EACH OUTCOME EXPLORE HOW IT COULD BE MEASURED

THIS MIGHT HELP TO REDUCE THE NUMBER OF OUTCOMES FURTHER

Let's talk about XXX in more depth. How would you describe a good outcome for XXX, and how would you describe a not so good outcome? If you had to come-up with a four point scale, how would you express each point on that scale in words?

OK TO LEAD A BIT HERE IF NECESSARY

How would you group these outcomes? THIS MIGHT ALSO HELP TO REDUCE THE NUMBER OF OUTCOMES FURTHER

Trust (15 mins)

Do you trust health and biomedical researchers/funders to achieve the sorts of things we've discussed tonight? Why?

READ OUT EACH STATEMENTS BELOW AND GET SHOW OF HANDS RESPONSE AS TO WHO DISAGREES AND WHO AGREES. THEN EXPLORE REASONS FOR DIFFERENT OPINIONS.

DEPENDING ON TIME, TRY TO USE 4 STATEMENTS IN EACH GROUP. SELECT THOSE WHERE THE SENTIMENTS HAVE NOT YET BEEN EXPLORED/REVEALED

F1 – Concerns about science

1. The more I know about science the more worried I am
2. I don't understand the point of all the science being done today
3. Scientific advances tend to benefit the rich more than they benefit the poor

F2 – Enthusiasm about science

1. Government funding for science should be cut because the money can be better spent elsewhere
2. On the whole, science will make our lives easier
3. Scientists make a valuable contribution to society

F4 – Attitudes towards science in the economy (and science careers)<br>1. Scientific research makes a direct contribution to economic growth in the UK
F5 – Wider values and beliefs<br>1. We depend too much on science and not enough on faith

Close	
This project is a research grant sponsored by the Medical Research Council, which is the body that invests tax payers' money in biomedical and health research. The aim of the project is to understand how people value scientific research. There will be some more focus groups and we are doing interviews with researchers funded by MRC, then in the autumn there will be online surveys of MRC funded researchers and members of the public.<br>Thanks very much for coming today.	

Wave 2 Topic Guide

Introduction (5 mins)	6.30-6.35/8.15-8.20
	Introduce self
	Introduce anyone else who is observing or helping
	Has anyone been to anything like this before?
	I have here a list of things I'd like to cover but really want to hear your views on the issues we'll be introducing.
	There are no right or wrong answers. Everyone is entitled to their own view, so I'd like to hear from everyone because everyone's view is valid.
	You don't have to answer all of the questions.
	You are free to leave before the end of the session, if you wish.
	I would like to audio record the discussions, just to save me taking notes, so I can listen to what you're all saying.
	No one will be identified in the report. All the information will be collected together and anonymised.
	This is just one session of 4 that we are running around the country on this project.
	Is everyone happy for me to record the session?
	Please could everyone turn off their mobile phone.
	SWITCH ON RECORDER
	You have been invited here today by a market research company. They are a separate organisation and we have just used them to find the sort of people we wanted to come along.<br>I want to stress that this is not research for marketing or product development. This is academic research and is being done purely to better understand how people understand

	health and medical research.<br><br>This project is a research grant sponsored by the Medical Research Council, which is the government body that invests tax payers' money in medical and health research. Decisions on which projects to fund are made by researchers who are experts in their fields.<br><br>The aim of the project is to understand how different people value scientific research.<br><br>As well as four focus groups and we are doing interviews with researchers funded by MRC, then in the autumn there will be online surveys of MRC funded researchers and 1,000 members of the public.

Round robin (5 mins) — 6.35-6.40/8.20-8.25

OK, let's go round and introduce everyone. Please just give your first name, and a little bit about yourself.

Defining research (5 mins) — 6.40-6.45/8.25-8.30

First I'd like to do a bit of brain storming.

What comes to mind if I say 'health research'? LIST ON FLIP CHART

What comes to mind if I say 'medical research'? LIST ON FLIP CHART

TRY TO SUM UP DIFFERENCES AND SIMILARITIES AND GET PARTICIPANTS TO AGREE THAT RESEARCH IS SOMETHING ALONG THE LINES OF:

**Studying something so that we (as humankind) can understand better how it works.**
**Biology: understanding how bodies work, physical sciences: understanding how the natural world works, social science: understanding how societies work, humanities: understanding how cultures work.**

How would this definition apply to health and biomedical research?

Research outcomes (20 mins) — 6.45-7.05/8.30-8.50

Pairs exercise (5 mins of the 20 mins)

To get their research ideas funded researchers in universities and research institutes and sometimes in private companies, have to put in proposals. I'd like you to get into pairs and spend a few minutes talking about how you would decide whether or not to fund a piece of research. What would you want to be told about the research in order to make a decision on whether or not to fund it?

GO ROUND AND GET EACH PAIR TO SAY WHAT THEY WANTED TO KNOW.

Did you consider:

- How the research would be conducted?
- What would give you confidence that the research might/would be successful?
- Who would conduct the research?
    - What type of organisation would they be based in?
    - Where, geographically, would they be based?

Outcomes – health/economic/scientific

What sort of outcomes do you think the people/organisations who do research and fund research are looking for/hope to achieve?

PROBE FOR MEDICINES AND PROCEDURES/TREATMENTS

FOCUS ON STATE FUNDED RESEARCH, RATHER THAN DRUGS, IF POSSIBLE

Are there other types of outcomes for other people? PROBE AS NECESSARY TO STIMULATE IDEAS: Patients, researchers, the public, children, elderly people, carers, doctors, nurses, private companies, general public, trainees.

In reality where do you think the money would come from? What sorts of organisations would fund health and medical research?

HAVE 3 FLIP CHARTS HEADED: HEALTH, ECONOMIC, SCIENTIFIC PREPARED IN ADVANCE

AS OUTCOMES COME OUT OF THE DISCUSSION GET THE GROUP TO ALLOCATE IT TO ONE OF THESE 3 HEADINGS

FOR EACH CATEGORY PROBE FOR MORE OUTCOMES AND SUGGEST OTHERS FOR AGREEMENT AS APPROPRIATE

What about (other) health outcomes? E.g. new companies, new jobs

What about (other) economic outcomes? E.g. certain diseases of types of health problems

What about (other) scientific outcomes? E.g. improvements in knowledge that may be important for further research or that may lead to cures or information on prevention. How do scientists find out about the findings of each other's work?

Categories and measures (35 mins)

7.05-7.45/8.50-9.30

We're now going to split into 2 groups, 1 group will work with me and the other with XXX. Then we'll come back together and compare the thoughts of the 2 groups.

We want you to imagine that you have half a million pounds to invest in some medical or health research project. There are several applications for this money, which has to be spent on just one project. All the applicants are professors in UK universities who are internationally known to be experts in their field. How would you decide which project to fund?

From the previous groups, plus some reading on the subject, we have a list of the outcomes people expect from research. In 2 groups we are going to work through each one to see whether it is important to you, whether you would say it is healthcare, economic or scientific in nature and how you would judge whether each outcome is good or poor.

1. Number of people affected by the disease	Is this an important criteria in deciding whether or not to fund a research project?<br>IF YES:<br>Would you categorise this as health/economic or scientific benefit?<br>POSSIBLE PROBES TO IDENTIFY LEVELS:<br>How many people would have to be affected by the disease for it to be important to do research? And how many would have to be affected to make it less important to do research?
2. Number of jobs created by the research project	Is this an important criteria in deciding whether or not to fund a research project?<br>IF YES:

	Would you categorise this as health/economic or scientific benefit?<br>POSSIBLE PROBES TO IDENTIFY LEVELS:<br>How many jobs would need to be created by the research project for this to be an important consideration in deciding whether or not to fund the research? And how many would have to be created for this to be an unimportant criteria?
3. Articles published in research journals	Is this an important criteria in deciding whether or not to fund a research project?<br>IF YES:<br>Would you categorise this as health/economic or scientific benefit?<br>POSSIBLE PROBES TO IDENTIFY LEVELS:<br>How many articles would you expect to be published from a good piece of research? And how many from a poor research project? How would you judge the quality of the articles?
4. Improved understanding of how the body works	Is this an important criteria in deciding whether or not to fund a research project?<br>IF YES:<br>Would you categorise this as health/economic or scientific benefit?<br>POSSIBLE PROBES TO IDENTIFY LEVELS:<br>What would you think would be a good level of improvement and what would not be much of an improvement at all?
5. Defence of the UK against bioterrorism	Is this an important criteria in deciding whether or not to fund a research project?<br>IF YES:<br>Would you categorise this as health/economic or scientific benefit?<br>POSSIBLE PROBES TO IDENTIFY LEVELS:<br>How much defence against terrorism do we need/should we have as a country? And what would be an insufficient level or defence?
6. Likelihood of a new drug or treatment or medical device being developed	Is this an important criteria in deciding whether or not to fund a research project?<br>IF YES:<br>Would you categorise this as health/economic or scientific benefit?<br>POSSIBLE PROBES TO IDENTIFY LEVELS:<br>Would the value of this outcome depend on the number of people affected by the disease(s) that would be treated? Or by the severity of the disease(s)?
7. Cheaper ways of delivering treatment or medicines to patients	Is this an important criteria in deciding whether or not to fund a research project?<br>IF YES:<br>Would you categorise this as health/economic or scientific benefit?

	POSSIBLE PROBES TO IDENTIFY LEVELS:<br>How much money would need to be saved for this to be of value? This could be expressed as a proportion of the cost rather than an absolute value.
8. More efficient ways of targeting the drug at the people most likely to be helped, i.e. finding the right drug for each individual quickly	Is this an important criteria in deciding whether or not to fund a research project?<br>IF YES:<br>Would you categorise this as health/economic or scientific benefit?<br>POSSIBLE PROBES TO IDENTIFY LEVELS:<br>How would you measure whether this is a valuable improvement? The number of people affected? The money saved? Reduction in side effects?
9. Number of people likely to be helped by any drug or treatment developed	Is this an important criteria in deciding whether or not to fund a research project?<br>IF YES:<br>Would you categorise this as health/economic or scientific benefit?<br>POSSIBLE PROBES TO IDENTIFY LEVELS:<br>Is this the same as the number of people affected by the disease?<br>IF NOT:<br>How many people would have to be helped for this to be a good outcome? And how many would have to be helped for it to be less valuable? Or would you make a value judgement based on the severity of the disease(s)?
10. Better diagnosis	Is this an important criteria in deciding whether or not to fund a research project?<br>IF YES:<br>Would you categorise this as health/economic or scientific benefit?<br>POSSIBLE PROBES TO IDENTIFY LEVELS:<br>What do you mean by better diagnosis? How much quicker or more accurate would diagnosis have to be for research to be valuable? And what sort of improvement would not be worth having?
11. Better healthcare	Is this an important criteria in deciding whether or not to fund a research project?<br>IF YES:<br>Would you categorise this as health/economic or scientific benefit?<br>How would you measure whether healthcare was better?
12. Better trained health staff in surgeries and hospitals	Is this an important criteria in deciding whether or not to fund a research project?<br>IF YES:<br>Would you categorise this as health/economic or scientific benefit?<br>POSSIBLE PROBES TO IDENTIFY LEVELS:

	What impact would you expect better trained staff to have if they were very much better trained? And what about if they were only slightly better trained?
13. Better trained researchers	Is this an important criteria in deciding whether or not to fund a research project?<br>IF YES:<br>Would you categorise this as health/economic or scientific benefit?<br>POSSIBLE PROBES TO IDENTIFY LEVELS:<br>What impact would you expect better trained researchers to have if they were very much better trained? And what about if they were only slightly better trained?
14. Maintain or improve the ability to do research on the topic in the UK	Is this an important criteria in deciding whether or not to fund a research project?<br>IF YES:<br>Would you categorise this as health/economic or scientific benefit?<br>POSSIBLE PROBES TO IDENTIFY LEVELS:<br>How important is it that the UK can do research in a specific topic?
15. Provide information for people to improve their health	Is this an important criteria in deciding whether or not to fund a research project?<br>IF YES:<br>Would you categorise this as health/economic or scientific benefit?<br>How would you determine whether providing information is valuable? Would it be the provision of the information itself? Whether it's likely that people would take notice and change their behaviour? Whether after a period of time some (how many?) people have changed their behaviour?
16. Generation of new knowledge leading to more research	Is this an important criteria in deciding whether or not to fund a research project?<br>IF YES:<br>Would you categorise this as health/economic or scientific benefit?<br>POSSIBLE PROBES TO IDENTIFY LEVELS:<br>How much or what sort of new knowledge would be valuable? And what would be at the other end of the spectrum?
17. UK becomes centre for this type of research	Is this an important criteria in deciding whether or not to fund a research project?<br>IF YES:<br>Would you categorise this as health/economic or scientific benefit?<br>POSSIBLE PROBES TO IDENTIFY LEVELS:<br>How important is it that the UK is an international centre for some types of health and medical research? How would you know if we are thought of as an international centre? What sorts of indicators would you be looking for?

	And what would tell you that the UK is not an important centre for a specific type of health or medical research?
18. Better policy-making	Is this an important criteria in deciding whether or not to fund a research project?<br><br>IF YES:<br><br>Would you categorise this as health/economic or scientific benefit?<br><br>POSSIBLE PROBES TO IDENTIFY LEVELS:<br><br>How would you know that the research had informed policy-making? Would it be important to inform it at a regional, national or international level?
Give the discussion we've just had, do you want to add anything else to this list?	
Taking each of these we would like you to consider whether or not you would include it in your consideration of whether or not to fund a research project and the reason for your decision.	
If you would include it, we'd like you to say what would be a good outcome and what would be a bad outcome. For example, if you think that creating jobs is a criteria you would consider, how many jobs would you expect to be created for it to be a good outcome and how many would be a poor outcome.<br><br>If there is a criteria that you think is the same as something else, then say why you think that and if everyone agrees, we can move on to the next one.	
FACILITATORS MUST KEEP THE DISCUSSIONS MOVING THROUGH THE LIST AND ENSURE THAT THEY UNDERSTAND WHY A CRITERIA IS INCLUDED OR EXCLUDED FROM THE DECISION-MAKING.	
THE FACILITATOR MUST THEN MAKE SURE THAT FOR EACH CRITERIA THEY UNDERSTAND HOW IT WOULD BE MEASURED/ASSESSED AND WHY	
Categories and measures group discussion (15 mins)	7.45-8/9.30-9.45
BRING THE GROUP BACK TOGETHER AND WORK THROUGH EACH CRITERIA COMPARING RESPONSES	
Close	8pm/9.45pm
Thanks very much for coming today.	

Recruitment questionnaire

Briefing

We want to recruit four groups, each to comprise 5 men and 5 women for 8 to attend on the night. Each group will last 1½ hours.

Groups 1 and 2 to be held in north London/Hertfordshire/Essex borders, on Thursday 3 April, to start at 7pm.

Group 1 Participants must be aged 25-45 and from social grades ABC1

Group 2 Participants must be aged 46-70 and from social grades C2DE

Groups 3 and 4 to be held in Manchester/Leeds or similar northern town, on Tuesday 6 May, to start at 6.30pm.

Grou 3 Participants must be aged 25-45 ABC1 and from social grades C2DE

Group 4 Participants must be aged 46-70 and from social grades ABC1.

Please ensure a spread of ages in all the groups.

Introduction

Hello my name is… and I work for…… We are looking for people to come to a discussion about health research. Could you spare me a few minutes to answer some questions please?

Q1	SHOW CARD A<br>Do you or any of your close relatives work in any of the following occupations?		
	Market research	1	CLOSE
	Journalism	2	
	Public relations	3	
	Marketing	4	
	Scientific research	5	
	Doctor	6	
	Nurse	7	
	Dentist	8	
	Optician	9	
	Alternative medicine	10	
Q2	Are you…		
	Working full time	1	

	Working part-time	2	
	Retired/not working	3	
	Unemployed	4	CODE AS E
	Student	5	CODE AS C1
Q3	Job Title (WRITE IN)		
	Job Description (WRITE IN)		
	Size of Company (WRITE IN)		
	Qualifications (WRITE IN)		
	How many people are you responsible for? (WRITE IN)		
	CODE SOCIAL GRADE		
	A	1	REFER TO QUOTA
	B	2	
	C1	3	
	C2	4	
	D	5	
	E	6	
Q4	MALE	1	REFER TO QUOTA
	FEMALE	2	
Q5	What was your age last birthday?		
	25-45	1	REFER TO QUOTA

	46-70	2	
QA.	Have you EVER attended a group discussion or depth interview before?		
	Yes	1	ASK QF
	No	2	RECRUIT
QB.	Have you been to a group discussion or depth interview in the last 6 months?		
	Yes	1	CLOSE
	No	2	GO TO QG
QC.	How many group discussions/depth interviews have you been to in the last 2 years? (i.e. 6 months - 2 years ago)		
	None	1	GO TO QI
	1 or 2	2	GO TO QH
	More than 2	3	CLOSE
QD.	Did you go to any groups/depths between 2 and 7 years ago?		
	Yes	1	CLOSE
	No	2	GO TO QI
QE.	What was the subject of the discussion group(s)/depths you took part in in the past?<br>(WRITE IN SUBJECT MATTER AND APPROX - WHEN IT WAS FOR EACH OCCASION).		
	**IF ABOUT HEALTH OR RESEARCH - CLOSE**. THIS IS VERY IMPORTANT. THE RESPONDENT MUST NEVER HAVE PARTICIPATED IN A DISCUSSION ON THE SAME SUBJECT. OTHERWISE RECRUIT.		

	If you have any queries at all, please call your Manager<br>• At least half of each group/set of depths must be **brand new** recruits.<br>• The remaining half can have attended up to a maximum of 2 groups/depths in the last 2 years (i.e. 6 months -2 years ago)<br>• Those who have been to 2 groups/depths in the last 2 years must have had a 5 year gap before that<br>• **None** to have attended any group/depths in **last 6 months**<br>• **None ever** to have attended a group/depths on HEALTH OR SCIENTIFIC RESEARCH
	On recruitment, the respondent must be told:<br><br>"The session you will be attending will be audio recorded. The recording is primarily to ensure that there is a full record of the conversations and to save the researchers having to take extensive notes. In any report of the research material will be anonymised, so individual comments will remain confidential."<br><br>Participants should be given the date, time and location of the event in writing and the recruiter's contact details in case they have queries or need to cancel.

Interviews with researchers

Individuals were selected randomly from the list of researchers provided by the MRC, but with two constraints: (i) no more than two researchers from the same institution would be interviewed; and (ii) where two researchers from the same institution were selected, they should be from different departments. After selection, the sample was reviewed to ensure that it was suitably diverse; no amendments to it were made at this point. The initial nine researchers interviewed were based in seven different institutions and worked in the following fields: medical sociology, HIV and STIs, genetics, medical statistics, parasitology, epidemiology, pharmacology of inflammation, basic virology and neurobiology. They also covered a range of career stages. While we had originally planned to conduct 24 interviews, responses were fairly consistent across the initial set of nine, with little new content being suggested. Given this, it was decided that the remaining interviews would not be conducted at that stage, allowing more cognitive testing of the draft survey subsequently. The interview protocol is provided below.

Researchers interviewed were broadly in agreement with the draft framework developed from the literature, both in terms of its overall domains and the specific impacts within them. However, the following items were considered less important by at least one interviewee (reasons given in brackets):

- Obtaining future funding (not considered to be impact)
- Esteem measure (not considered to be impact)
- Wider participation (meaning not clear/ambiguous)
- Benefits to companies (considered to overlap with other items)
- Collaboration and networking (considered to be a facilitator not an impact)

It was also suggested by interviewees that we consider adding a statement on more effective targeting of future research and another relating to dissemination of knowledge.

Researcher interview protocol

Introduction

Over the past 15 years there has been a growing focus on measuring the impact arising from public investment in research and development. As part of their research funding applications, researchers often submit a ‘Pathway to Impact’ document, which is peer reviewed by referees and panel members. Similarly, the Funding Council assesses impact using a case study approach as part of the Research Excellence Framework, in which case studies are reviewed by academic peers and non-academic experts. However, in reaching an assessment reviewers cannot currently draw on comprehensive evidence of the views of beneficiaries (i.e. the general population) or the producers of research (i.e. biomedical and health researchers) to qualify or justify their recommendations. To address these issues, this study aims to understand the relative value that these two groups of stakeholders place on different types of research impact and to test the appropriateness of an established survey-based methodology known as Best-Worst Scaling to elicit such a valuation.

In the Best-Worst Scaling exercise, survey participants will be asked to consider a list of research outcomes and select which they judge represents the 'most important' impact and which represents the 'least important' impact. By repeating this and altering the research outcomes presented on each occasion, then combining data across the whole sample, we will build up information to create an overall 'importance' ranking of the full set of research outcomes.

Exploring the perceived value of different impacts will help inform the Research Councils and Funding Councils in the UK on their future policies on research impact, the assessment of which is being widely discussed in science policy circles internationally. The findings from this study are likely to contribute to those discussions, and may ultimately inform the way in which peer review panels consider different types of anticipated impact described in funding applications.

The aim of this stage is to help us design the survey instrument.

<u>Questions:</u>

Background

1. What is your broad area of research?
2. Would you classify yourself as a basic or clinical researcher?
3. How long have you been working in research? Would you classify yourself as early, mid or late career?

What is research impact?

4. What do you understand by research impact?
5. In your field, what kinds of impact might research produce?
6. What impacts do you, as a researcher, want to produce?
7. What impacts are funders looking for?
8. What impacts are actually produced in reality?
9. Probe any of the following that they didn't mention. What about:
    a. New knowledge produced
    b. Number of publications
    c. Quality measures for publications
    d. Funding for research
    e. Esteem measures
    f. Number of researchers trained
    g. Quality of researchers trained
    h. Collaboration and networking
    i. Wider participation in research
    j. Products and process developed
    k. Products and processes patented
    l. Products and processes licensed
    m. Products and processes used
    n. New businesses (spinouts)
    o. Benefits to companies
    p. Job creation
    q. Workforce development and increased economic competitiveness
    r. Impact on guidelines/policy in health

s. Impact on professional training or development in health
t. Impact on practice including saving NHS money, making processes more efficient or resilient etc.
u. impact on health and wellbeing (of a group of patients, or more widely)
v. Changes to policy outside of health
w. improvements in education
x. improvements in national security
y. improvements in international development
z. improvements in the environment
aa. improvements in the delivery of public services (outside of health) other/general
bb. Benefits to public wellbeing/society resulting from changes in policy/public services (outside health)
cc. Number and range of dissemination and outreach activities
dd. increased public understanding of and engagement with science

Classification of impacts

10. How might you think about grouping these different types of impact?
11. If possible, send classification – ask them what they think about it.
    a. Does it make sense?
    b. Are there overlaps between groups?
    c. Are there other ways you would think about grouping these impacts?

Rating of impacts

12. Which impacts do you think are most important?
    a. Why?

References

1. Castell S, Charlton A, Clemence M et al. *Public Attitudes to Science 2014: Main Report*. London, UK: Ipsos MORI 2014.
2. Evans G, Durant J. The relationship between knowledge and attitudes in the public understanding of science in Britain. *Public Understanding of Science* 1995; 4(1), 57-74.
3. Owens C, Ley A, Aitken P. Do different stakeholder groups share mental health research priorities? A four-arm Delphi study. *Health Expectations* 2008; 11(4), 418-431.
4. Miller JD. Public Understanding of, and Attitudes toward, Scientific Research: What We Know and What We Need to Know. *Public Understanding of Science* 2004; 13(3), 273-294.
5. Office of Science and Technology, the Wellcome Trust. *Science and the public. A review of science communication and public attitudes to science in Britain*. London: The Wellcome Trust 2000.

Frameworks:

6. **Snowball Metrics: http://www.snowballmetrics.com/** [Accessed: 26 November 2015]
7. **Canadian Academy of Health Sciences (CAHS):** CAHS. *Making an impact. A preferred framework and indicators to measure returns on investment in health research*. Ottawa, ON: Canadian Academy of Health Sciences 2009.
8. **Excellence in Research for Australia:** http://www.arc.gov.au/excellence-research-australia [Accessed: 26 November 2015]
9. **National Institute for Health Research dashboard:** El Turabi A et al. A Novel Performance Monitoring Framework for Health Research Systems: Experiences of the National Institute for Health Research in England. *Health Research Policy and Systems*,2011; Vol. 9, p. 13.
10. **Research Excellence Framework:** http://www.ref.ac.uk/ [Accessed: 26 November 2015]
11. **SIAMPI:** http://siampi.eu/ [Accessed: 26 November 2015]
12. **Agence d'évaluation de la recherche et de l'enseignement supérieur (AERES):** http://www.aeres-evaluation.com/ [Accessed: 26 November 2015]
13. **Research Councils UK Outcomes System (ROS):** http://www.esrc.ac.uk/_images/Output_types_on_ROS_tcm8-14587.pdf (as of 12 March 2014)
14. **Payback Framework:** Buxton M, Hanney S. How can payback from health services research be assessed? *Journal of Health Services Research and Policy* 1996; 1, 35-43.
15. **National Institutes of Health (NIH):** Definitions of Criteria and Considerations for Research Project Grant (RPG/R01/R03/R15/R21/R34) Critiques. http://grants.nih.gov/grants/peer/critiques/rpg.htm [Accessed: 26 November 2015]
16. **National Science Foundation (NSF):** Grant Proposal Guide. http://www.nsf.gov/pubs/policydocs/pappguide/nsf16001/gpg_index.jsp?org=NSF [Accessed: 26 November 2015]
17. **Consortia Advancing Standards in Research Administration Information (CASRAI):** http://casrai.org/ [Accessed: 26 November 2015]
18. **Researchfish**: https://www.researchfish.com [Accessed: 26 November 2015]
