# Supplementary material for: Understanding the relative valuation of research impact: a best–worst scaling experiment of the general public and biomedical and health researchers
Source: BMJ Open. 2016 Aug 18;6(8):e010916. doi: 10.1136/bmjopen-2015-010916 (PMC5013498; doi:10.1136/bmjopen-2015-010916)
Supplement: Supplementary file [file bmjopen-2015-010916supp3.pdf]

Supplementary File 3: Constructing and testing the survey instrument

Further details of the cognitive interviews and pilot used to test the surveys are provided below.

Cognitive interviews

The draft questionnaire along with attributes and levels were further refined in cognitive interviews. Cognitive interviews are a structured, systematic interview technique used to understand the cognitive processes respondents use when interpreting and responding to questions. These interviews aimed to test the complete survey in terms of its wording and layout, and identify any aspects which might be considered ambiguous or cause confusion. It was also possible to get an estimate of survey response time from cognitive interviews.

Interviews were conducted with six members of the public and two researchers. Researchers were selected at random from the MRC list previously used for identifying researchers for the earlier interviews. On this occasion, for logistical reasons, one researcher was selected from a London institution and one from a Cambridge institution. It was ensured that the two worked in different fields. Members of the public were recruited by Plus Four Market Research according to the same criteria as the focus groups in terms of previous participation in similar exercises and occupations to exclude. The final sample was made up of three men and three women, with ages ranging from 21 to 65 and social grades from B to D. Interviews were conducted in Cambridge in August 2014.

In each interview we asked the respondent to complete the BWS exercise (up to six questions, each on a separate sheet) and the appropriate preceding section of the survey (background questions on their work for researchers and PAS views on science questions for the general public). Respondents completed the survey reading and thinking aloud, after which interviewers asked specific questions on different elements of the survey, focusing particularly on the interpretation of words and phrases. The topic guide, including instructions given to interviewees and probe questions used by interviewers, is provided below.

The cognitive interviews proved valuable in refining the structure and wording of the survey instrument. Overall, the terms 'important', 'research' and 'biomedical and health' all seemed fairly well understood. When interviewees understood all of the statements presented, they often described using a combination of 'heart and head' to decide which was most important. Some also commented that the least important was judged more on 'gut instinct', and in some instances this appeared to be due to not understanding some of the statements. To aid understanding, the following changes were made to the general structure as a result of the interviews:

- The number of statements per question was reduced. One of the aims of conducting the interviews was to establish which of the thirteen potential items were most consistently understood, so that a smaller set could be used in the final survey. Interviewees generally felt that around eight statements was a manageable number.
- The length of the statements was reduced where possible, since some interviewees stopped reading part-way through, particularly when they reached a word that triggered a value

judgement. For example, “terminally ill patient” was generally deemed important, while “company” was generally considered unimportant, regardless of the following part of the sentence.

- Statements were ordered randomly in each question. Interviewees confirmed our concern that position in the list might bias decisions and that maintaining the same order throughout might lead to participants not noticing subtle changes in statements from one question to the next.
- Wording was made more varied, where possible, although it was also important to ensure that statements within a domain were still comparable. Sometimes minor variations in similar statements were missed by interviewees, while others felt they were being tested on spotting the changes from one question to the next.
- Ordering of sections of the survey was changed, so that the BWS task was presented before the general questions or PAS questions, as in some instances these were found to influence responses on the BWS task.

Specific observations on each domain led to some being dropped entirely (to arrive at our final set of eight) and others being reworded to ensure that they were clear and consistently understood. Problematic, emotive or ambiguous words were also identified and replaced with alternatives where possible (e.g. reference to “practice” was considered too vague, “terminally ill patient” was too emotive). These changes led to the development of the pilot version of the survey instrument, as set out in Supplementary File 4. PAS statements were not changed, as the analysis required that they remained identical to those used in the PAS survey.

Topic Guide – General Public

Introduction

[Give cash incentive at the start and ask to sign sheet]

My name is XX and I'm a researcher from RAND Europe. RAND Europe is a not-for-profit research organisation. Before we start, I just want to explain what we're doing here today. We are doing a research project at the moment in which we are asking people to fill in a survey asking for their views on medical and health research, and the benefits that can come from it. Before we launch the survey we want to test out the questions and see how people understand them, and so that's what we would like your help with today.

What we would like you to do is have a go at filling out the survey questionnaire, as you would if it was sent to you. So if there are parts you would read, then go ahead and take your time reading them, but if there are parts you would skim over or skip, then please also do that. The really important thing is that while you are working through the questionnaire, I would like you to say out loud whatever it is you are reading or thinking – so just all the thoughts that come into your head as you go through.

There are no right or wrong answers at all – we are just interested in how people find filling in the survey. I also just want to emphasise that everything will be entirely confidential. If it's ok with you, I'd like to record the session, just so that I don't have to take detailed notes and can pay full attention to what you're saying. We'll destroy the recordings at the end of the study – it's only to help us remember the important points for designing the survey and, as I said, everything will be kept strictly confidential. Is that ok?

Do you have any questions before we begin?

Probes

Spontaneous

- I noticed you were spending some time with that question, can you tell me what you were thinking about?
- I noticed you hesitated before you answered – what were you thinking about?
- You answered that very quickly – why was that?

Section 1 – Introduction and views on science

Use spontaneous probes, don't spend too long on this bit.

Section 2 – Best Worst Scaling exercise

1. Intro probes

- Can you describe what sort of research you were thinking about when answering these questions?
- What do you understand by biomedical and health research?
- What did you understand by 'important' here?
- How did you go about answering the question?
- How did you arrive at your final answer?
- Was it easy or difficult to answer? Why was that?

2. Probes on specific impact statements (see individual statements below)

Section 2: BWS exercises

Sheet 1

- What did you understand by 'important' here?
- How did you go about answering the question?
- How did you arrive at your final answer?
- Was it easy or difficult to answer? Why was that?

I'd like to go through each statement and what you understood by it so we can check the wording of them.

The research replicates the work of others, helping to strengthen the evidence of how some things work

- Looking at the first statement, what did you understand by that?
- Could you repeat the statement, using your own words?

The research generates knowledge that is world-leading in terms of originality, significance and rigour

- Looking at the second statement, what did you understand by that?
- What does 'world leading' mean to you?

Research helps train scientists who go on to be world-leading in terms of originality, significance and rigour

- Looking at the third statement, what does that mean to you?

The research supports the training of future scientists that end up applying their skills in science based industries

- What do you understand by the fourth statement?
- What does 'science-based industries' mean to you?

The research leads to the establishment of companies that create a substantial number of new jobs in regions where universities are based

- What do you understand by the fifth statement?
- What does 'a substantial number' mean to you?
- What type of jobs were you thinking about?
- What did you understand by 'regions where universities are based'?

The research produces a discovery that forms the basis a company that attracts significant amounts of funding from investors [in exchange for a large number of shares]

- What do you understand by the sixth statement?
- What type of discovery were you thinking of?
- What type of company were you thinking of?
- What does 'significant amounts of funding' mean to you?
- What, to you, are 'investors'?
- What do you understand by 'a large number of shares'?

Research contributes to an overseas company deciding to move a major part of its operations to the UK

- What do you understand by the next statement?
- What did you understand by ‘a major part of its operations’?

Research produces a discovery which is the foundation of a new company

- What do you understand by the next statement?
- What type of discovery were you thinking of?
- What type of company were you thinking of?

Research contributes to an additional three years of life for a terminally ill patient free of disability

- What do you understand by the next statement?
- What does ‘free of disability’ mean to you?

Findings from the research influence practice nationally

- What does the next statement mean to you?
- What do you understand by the work ‘practice’?
- What does ‘nationally’ mean to you?

Research contributes to a recommendation that a new treatment may offer advantages over existing treatments

- What does the next statement mean to you?
- What do you understand by a ‘recommendation’ here?
- What does ‘treatment’ mean to you?

Research contributes to better care being provided at a slightly higher cost

- What does the next statement mean to you?
- What do you understand by ‘better care’?
- What does ‘a slightly higher cost’ mean to you?

Researchers give interviews to the media about their research

- What did you understand by this statement?
- What type of media were you thinking of?
- Did you think about this happening once or more than once? If more than once, how frequently?

Sheet 2

- How did you go about answering the question?
- Was it easy or difficult to answer? Why was that?

The research brings together the works of a range of others, identifying areas of consistency and difference

- Looking at the first statement, what did you understand by that?
- Could you repeat the statement, using your own words?

The research generates knowledge that is internationally excellent in terms of originality, significance and rigour

- Looking at the second statement, what did you understand by that?
- What does 'internationally excellent' mean to you?

Research helps train scientists who go on to be internationally excellent in terms of originality, significance and rigour

- Looking at the third statement, what does that mean to you?

The research supports the training of future scientists that end up working in the healthcare system

- What do you understand by the fourth statement?
- What does 'the healthcare system' mean to you?

The research leads to the establishment of companies that create a small number of new jobs in universities

- What do you understand by the fifth statement?
- What does 'a small number' mean to you?
- What type of jobs were you thinking about?

The research produces a discovery that forms the basis of a small start-up company owned by the researchers

- What do you understand by the sixth statement?
- What type of discovery were you thinking of?
- What type of company were you thinking of?
- What, to you, is 'a small start-up company'?

Research contributes to an overseas company deciding to set up a new UK research centre

- What do you understand by the next statement?
- What did you understand by 'a new UK research centre'?

Research produces a discovery worth protecting with a patent

- What do you understand by the next statement?
- What type of discovery were you thinking of?
- What do you understand by 'protecting with a patent'?

Research contributes to an additional month of life for a terminally ill patient free of disability

- What do you understand by the next statement?

Findings from the research influence practice internationally

- What does the next statement mean to you?
- What does 'internationally' mean to you?

Research contributes to a recommendation that a new treatment may be as good as an existing treatment

- What does the next statement mean to you?
- Can you repeat the statement in your own words?

Research contributes to care being provided more cheaply without any change in quality

- What does the next statement mean to you?
- What do you understand by 'care'?
- What does 'more cheaply' mean to you?
- What does 'quality' mean to you?

Researchers give interviews to the media about their research

{NO QUESTIONS, ALREADY COVERED IN SHEET 1}

Sheet 3

The research replicates the work of others, helping to strengthen the evidence of how some things work

{NO QUESTIONS, ALREADY COVERED IN SHEET 1}

The research generates knowledge that is internationally excellent in terms of originality, significance and rigour
{NO QUESTIONS, ALREADY COVERED IN SHEET 2}

Research helps train scientists who go on to be recognised nationally in terms of originality, significance and rigour

- Looking at this statement, what did you understand by that?
- What does 'recognised nationally' mean to you?

The research supports the training of future scientists that end up applying their skills in science based industries
{NO QUESTIONS, ALREADY COVERED IN SHEET 1}

The research leads to the establishment of companies that create a small number of new jobs in universities
{NO QUESTIONS, ALREADY COVERED IN SHEET 2}

The research produces a discovery that forms the basis a company that is acquired [bought] by a major multinational [pharmaceutical] company

- What do you understand by the next statement?
- What type of discovery were you thinking of?
- What type of company were you thinking of?

Research contributes to an overseas company deciding to move a major part of its operations to the UK
{NO QUESTIONS, ALREADY COVERED IN SHEET 1}

Research produces a discovery worth protecting with a patent
{NO QUESTIONS, ALREADY COVERED IN SHEET 2}

Research contributes to an additional year of life for a terminally ill patient free of disability

- What do you understand by this statement?

Findings from the research influence practice in the local hospital

- What does the next statement mean to you?
- What do you understand by 'the local hospital'?

Research contributes to a recommendation that a new treatment should entirely replace an existing treatment

- What do you understand by this statement?

Research contributes to better care being provided at very much higher cost

- What does the next statement mean to you?
- What does 'very much higher cost' mean to you?

Researchers talk in schools about their research

- What do you understand by this statement?
- What kind of schools were you thinking about?
- What types of events were you thinking about?
- Did you think about this happening once or more than once? If more than once, how frequently?

Sheet 4

The research results in a new observation or discovery, helping to focus on subsequent research activities

- Looking at the first statement, what did you understand by that?
- Could you repeat the statement, using your own words?

The research generates knowledge that is recognised internationally in terms of originality, significance and rigour

- Looking at the second statement, what did you understand by that?
- What does 'recognised internationally' mean to you?

Research helps train scientists who go on to be recognised nationally in terms of originality, significance and rigour

{NO QUESTIONS, ALREADY COVERED IN SHEET 3}

The research supports the training of future scientists that take their knowledge of science into a range of other professions such as teaching, business and civil service

- What do you understand by the fourth statement?
- What sort of professions were you thinking about?

The research leads to the establishment of companies that create a substantial number of new jobs across the UK

- What do you understand by the fifth statement?
- What does 'a substantial number' mean to you?
- What type of jobs were you thinking about?
- What did you understand by 'across the UK'?

The research produces a discovery that forms the basis a company that is acquired [bought] by a major multinational [pharmaceutical] company

{NO QUESTIONS, ALREADY COVERED IN SHEET 3}

Research contributes to an overseas company deciding to partly fund an existing UK research centre

- What do you understand by the next statement?
- What did you understand by 'a UK research centre'?
- What does 'partly fund' mean to you?
- What type of company were you thinking of?

Research produces a discovery which a company pays to use

- What do you understand by the next statement?
- What type of discovery were you thinking of?
- What type of company were you thinking of?

Research contributes to an additional year of life for a terminally ill patient free of disability

- What do you understand by this statement?

Findings from the research influence practice in the local region

- What does the next statement mean to you?
- What do you understand by 'the local region'?

Research contributes to a recommendation that a new treatment should not be used

- What do you understand by this statement?
- What sort of treatment were you thinking of?

Research contributes to better care being provided at the same cost

- What does the next statement mean to you?

Researchers give interviews to the media about their research
{NO QUESTIONS, ALREADY COVERED IN SHEET 1}

Sheet 5

The research shows that something does not work (i.e. it is a negative finding), eliminating the need for further investigation

- Looking at the first statement, what did you understand by that?
- Could you repeat the statement, using your own words?

The research generates knowledge that is recognised nationally in terms of originality, significance and rigour

- Looking at the second statement, what did you understand by that?
- What does 'recognised nationally' mean to you?

Research helps train scientists who go on to be recognised internationally in terms of originality, significance and rigour

- Looking at the second statement, what did you understand by that?
- What does 'recognised internationally' mean to you?

The research supports the training of future scientists that end up undertaking research in universities

- What do you understand by the fourth statement?

The research leads to the establishment of companies that create a small number of new jobs in the town where universities are based

- What do you understand by the fifth statement?
- What does 'a small number' mean to you?
- What type of jobs were you thinking about?
- What did you understand by 'the town where universities are based'?

The research produces a discovery that forms the basis of a start-up company that attracts small amounts of seed funding from investors [in exchange a small number of shares]

- What do you understand by the next statement?
- What type of discovery were you thinking of?
- What type of company were you thinking of?
- What do you understand by 'seed funding'?
- What type of investors were you thinking of?

Research contributes to an overseas company deciding to fund a follow-up study in the UK

- What do you understand by the next statement?
- What type of company were you thinking of?
- What do you understand by 'follow-up study'?

Research produces a discovery which is made freely available to companies and organisations and improves how they operate

- What do you understand by the next statement?
- What type of discovery were you thinking of?
- What types of companies and organisations were you thinking of?
- What types of improvements were you thinking of?
- What does 'freely available' mean to you?

Research contributes to an additional 6 months of life for a terminally ill patient free of disability

- What do you understand by this statement?

Findings from the research influence practice in the local hospital
{NO QUESTIONS, ALREADY COVERED IN SHEET 3}

Research contributes to a recommendation that a new treatment should entirely replace an existing treatment
{NO QUESTIONS, ALREADY COVERED IN SHEET 3}

Research contributes to better care being provided at very much higher cost
{NO QUESTIONS, ALREADY COVERED IN SHEET 3}

Researchers give interviews to the media about their research
{NO QUESTIONS, ALREADY COVERED IN SHEET 1}

Sheet 6

The research brings together the works of a range of others, identifying areas of consistency and difference
{NO QUESTIONS, ALREADY COVERED IN SHEET 2}

The research generates knowledge that is world-leading in terms of originality, significance and rigour
{NO QUESTIONS, ALREADY COVERED IN SHEET 1}

Research helps train scientists who go on to be recognised internationally in terms of originality, significance and rigour
{NO QUESTIONS, ALREADY COVERED IN SHEET 5}

The research supports the training of future scientists that end up working in the healthcare system
{NO QUESTIONS, ALREADY COVERED IN SHEET 2}

The research leads to the establishment of companies that create a substantial number of new jobs in regions where universities are based
{NO QUESTIONS, ALREADY COVERED IN SHEET 1}

The research produces a discovery that forms the basis of a start-up company that attracts small amounts of seed funding from investors [in exchange a small number of shares]
{NO QUESTIONS, ALREADY COVERED IN SHEET 5}

Research contributes to an overseas company deciding to set up a new UK research centre
{NO QUESTIONS, ALREADY COVERED IN SHEET 2}

Research produces a discovery which is the foundation of a new company
{NO QUESTIONS, ALREADY COVERED IN SHEET 1}

Research contributes to an additional 6 months of life for a terminally ill patient free of disability
{NO QUESTIONS, ALREADY COVERED IN SHEET 5}

Findings from the research influence practice in the local region
{NO QUESTIONS, ALREADY COVERED IN SHEET 4}

Research contributes to a recommendation that a new treatment should not be used
{NO QUESTIONS, ALREADY COVERED IN SHEET 4}

Research contributes to better care being provided at the same cost
{NO QUESTIONS, ALREADY COVERED IN SHEET 4}

Researchers talk in schools about their research
{NO QUESTIONS, ALREADY COVERED IN SHEET 3}

Wrap up

Thank you very much for taking the time to come in – it's been really helpful for us to hear your thoughts on the questions. As I mentioned at the start, we'll destroy the recording at the end of the study and your name won't be used in anything.

Are there any other questions you have now that we've finished?

Topic Guide – Researchers

Introduction

My name is XX and I’m a researcher from RAND Europe. RAND Europe is a not-for-profit research organisation. Before we start, I just want to explain what we’re doing here today. We are doing a research project at the moment in which we are asking people to fill in a survey asking for their views on medical and health research, and the benefits that can come from it. Before we launch the survey we want to test out the questions and see how people understand them, and so that’s what we would like your help with today.

What we would like you to do is have a go at filling out the survey questionnaire, as you would if it was sent to you. So if there are parts you would read, then go ahead and take your time reading them, but if there are parts you would skim over or skip, then please also do that. The really important thing is that while you are working through the questionnaire, I would like you to say out loud whatever it is you are reading or thinking – so just all the thoughts that come into your head as you go through.

There are no right or wrong answers at all – we are just interested in how people find filling in the survey. I also just want to emphasise that everything will be entirely confidential. If it’s ok with you, I’d like to record the session, just so that I don’t have to take detailed notes and can pay full attention to what you’re saying. We’ll destroy the recordings at the end of the study – it’s only to help us remember the important points for designing the survey and, as I said, everything will be kept strictly confidential. Is that ok?

Do you have any questions before we begin?

Probes

Spontaneous

- I noticed you were spending some time with that question, can you tell me what you were thinking about?
- I noticed you hesitated before you answered – what were you thinking about?
- You answered that very quickly – why was that?

Section 1 – Background

Use spontaneous probes, don’t spend too long on this bit.

For some of the quantitative questions ask:

- How did you work that out?
- How did you arrive at that answer?

Section 2 – Best Worst Scaling exercise

1. Intro probes

- Can you describe what sort of research you were thinking about when answering these questions?
- What do you understand by biomedical and health research?
- What did you understand by ‘important’ here?
- How did you go about answering the question?
- How did you arrive at your final answer?
- Was it easy or difficult to answer? Why was that?

2. Probes on specific impact statements (see individual statements below)

BWS exercises

Sheet 1

- What did you understand by 'important' here?
- How did you go about answering the question?
- How did you arrive at your final answer?
- Was it easy or difficult to answer? Why was that?

I'd like to go through each statement and what you understood by it so we can check the wording of them.

The research replicates the work of others, helping to strengthen the evidence of how some things work

- Looking at the first statement, what did you understand by that?
- Could you repeat the statement, using your own words?

The research generates knowledge that is world-leading in terms of originality, significance and rigour

- Looking at the second statement, what did you understand by that?
- What does 'world leading' mean to you?

Research helps train scientists who go on to be world-leading in terms of originality, significance and rigour

- Looking at the third statement, what does that mean to you?

The research supports the training of future scientists that end up applying their skills in science based industries

- What do you understand by the fourth statement?
- What does 'science-based industries' mean to you?

The research leads to the establishment of companies that create a substantial number of new jobs in regions where universities are based

- What do you understand by the fifth statement?
- What does 'a substantial number' mean to you?
- What type of jobs were you thinking about?
- What did you understand by 'regions where universities are based'?

The research produces a discovery that forms the basis a company that attracts significant amounts of funding from investors [in exchange for a large number of shares]

- What do you understand by the sixth statement?
- What type of discovery were you thinking of?
- What type of company were you thinking of?
- What does 'significant amounts of funding' mean to you?
- What, to you, are 'investors'?
- What do you understand by 'a large number of shares'?

Research contributes to an overseas company deciding to move a major part of its operations to the UK

- What do you understand by the next statement?
- What did you understand by ‘a major part of its operations’?

Research produces a discovery which is the foundation of a new company

- What do you understand by the next statement?
- What type of discovery were you thinking of?
- What type of company were you thinking of?

Research contributes to an additional three years of life for a terminally ill patient free of disability

- What do you understand by the next statement?
- What does ‘free of disability’ mean to you?

Findings from the research influence practice nationally

- What does the next statement mean to you?
- What do you understand by the work ‘practice’?
- What does ‘nationally’ mean to you?

Research contributes to a recommendation that a new treatment may offer advantages over existing treatments

- What does the next statement mean to you?
- What do you understand by a ‘recommendation’ here?
- What does ‘treatment’ mean to you?

Research contributes to better care being provided at a slightly higher cost

- What does the next statement mean to you?
- What do you understand by ‘better care’?
- What does ‘a slightly higher cost’ mean to you?

Researchers give interviews to the media about their research

- What did you understand by this statement?
- What type of media were you thinking of?
- Did you think about this happening once or more than once? If more than once, how frequently?

Sheet 2

- How did you go about answering the question?
- Was it easy or difficult to answer? Why was that?

The research brings together the works of a range of others, identifying areas of consistency and difference

- Looking at the first statement, what did you understand by that?
- Could you repeat the statement, using your own words?

The research generates knowledge that is internationally excellent in terms of originality, significance and rigour

- Looking at the second statement, what did you understand by that?
- What does ‘internationally excellent’ mean to you?

Research helps train scientists who go on to be internationally excellent in terms of originality, significance and rigour

- Looking at the third statement, what does that mean to you?

The research supports the training of future scientists that end up working in the healthcare system

- What do you understand by the fourth statement?
- What does ‘the healthcare system’ mean to you?

The research leads to the establishment of companies that create a small number of new jobs in universities

- What do you understand by the fifth statement?
- What does ‘a small number’ mean to you?
- What type of jobs were you thinking about?

The research produces a discovery that forms the basis of a small start-up company owned by the researchers

- What do you understand by the sixth statement?
- What type of discovery were you thinking of?
- What type of company were you thinking of?
- What, to you, is ‘a small start-up company’?

Research contributes to an overseas company deciding to set up a new UK research centre

- What do you understand by the next statement?
- What did you understand by ‘a new UK research centre’?

Research produces a discovery worth protecting with a patent

- What do you understand by the next statement?
- What type of discovery were you thinking of?
- What do you understand by ‘protecting with a patent’?

Research contributes to an additional month of life for a terminally ill patient free of disability

- What do you understand by the next statement?

Findings from the research influence practice internationally

- What does the next statement mean to you?
- What does 'internationally' mean to you?

Research contributes to a recommendation that a new treatment may be as good as an existing treatment

- What does the next statement mean to you?
- Can you repeat the statement in your own words?

Research contributes to care being provided more cheaply without any change in quality

- What does the next statement mean to you?
- What do you understand by 'care'?
- What does 'more cheaply' mean to you?
- What does 'quality' mean to you?

Researchers give interviews to the media about their research

{NO QUESTIONS, ALREADY COVERED IN SHEET 1}

Sheet 3

The research replicates the work of others, helping to strengthen the evidence of how some things work

{NO QUESTIONS, ALREADY COVERED IN SHEET 1}

The research generates knowledge that is internationally excellent in terms of originality, significance and rigour
{NO QUESTIONS, ALREADY COVERED IN SHEET 2}

Research helps train scientists who go on to be recognised nationally in terms of originality, significance and rigour

- Looking at this statement, what did you understand by that?
- What does 'recognised nationally' mean to you?

The research supports the training of future scientists that end up applying their skills in science based industries
{NO QUESTIONS, ALREADY COVERED IN SHEET 1}

The research leads to the establishment of companies that create a small number of new jobs in universities
{NO QUESTIONS, ALREADY COVERED IN SHEET 2}

The research produces a discovery that forms the basis a company that is acquired [bought] by a major multinational [pharmaceutical] company

- What do you understand by the next statement?
- What type of discovery were you thinking of?
- What type of company were you thinking of?

Research contributes to an overseas company deciding to move a major part of its operations to the UK
{NO QUESTIONS, ALREADY COVERED IN SHEET 1}

Research produces a discovery worth protecting with a patent
{NO QUESTIONS, ALREADY COVERED IN SHEET 2}

Research contributes to an additional year of life for a terminally ill patient free of disability

- What do you understand by this statement?

Findings from the research influence practice in the local hospital

- What does the next statement mean to you?
- What do you understand by 'the local hospital'?

Research contributes to a recommendation that a new treatment should entirely replace an existing treatment

- What do you understand by this statement?

Research contributes to better care being provided at very much higher cost

- What does the next statement mean to you?
- What does 'very much higher cost' mean to you?

Researchers talk in schools about their research

- What do you understand by this statement?
- What kind of schools were you thinking about?
- What types of events were you thinking about?
- Did you think about this happening once or more than once? If more than once, how frequently?

Sheet 4

The research results in a new observation or discovery, helping to focus on subsequent research activities

- Looking at the first statement, what did you understand by that?
- Could you repeat the statement, using your own words?

The research generates knowledge that is recognised internationally in terms of originality, significance and rigour

- Looking at the second statement, what did you understand by that?
- What does 'recognised internationally' mean to you?

Research helps train scientists who go on to be recognised nationally in terms of originality, significance and rigour

{NO QUESTIONS, ALREADY COVERED IN SHEET 3}

The research supports the training of future scientists that take their knowledge of science into a range of other professions such as teaching, business and civil service

- What do you understand by the fourth statement?
- What sort of professions were you thinking about?

The research leads to the establishment of companies that create a substantial number of new jobs across the UK

- What do you understand by the fifth statement?
- What does 'a substantial number' mean to you?
- What type of jobs were you thinking about?
- What did you understand by 'across the UK'?

The research produces a discovery that forms the basis a company that is acquired [bought] by a major multinational [pharmaceutical] company

{NO QUESTIONS, ALREADY COVERED IN SHEET 3}

Research contributes to an overseas company deciding to partly fund an existing UK research centre

- What do you understand by the next statement?
- What did you understand by 'a UK research centre'?
- What does 'partly fund' mean to you?
- What type of company were you thinking of?

Research produces a discovery which a company pays to use

- What do you understand by the next statement?
- What type of discovery were you thinking of?
- What type of company were you thinking of?

Research contributes to an additional year of life for a terminally ill patient free of disability

- What do you understand by this statement?

Findings from the research influence practice in the local region

- What does the next statement mean to you?
- What do you understand by 'the local region'?

Research contributes to a recommendation that a new treatment should not be used

- What do you understand by this statement?
- What sort of treatment were you thinking of?

Research contributes to better care being provided at the same cost

- What does the next statement mean to you?

Researchers give interviews to the media about their research
{NO QUESTIONS, ALREADY COVERED IN SHEET 1}

<u>Sheet 5</u>

The research shows that something does not work (i.e. it is a negative finding), eliminating the need for further investigation

- Looking at the first statement, what did you understand by that?
- Could you repeat the statement, using your own words?

The research generates knowledge that is recognised nationally in terms of originality, significance and rigour

- Looking at the second statement, what did you understand by that?
- What does 'recognised nationally' mean to you?

Research helps train scientists who go on to be recognised internationally in terms of originality, significance and rigour

- Looking at the second statement, what did you understand by that?
- What does 'recognised internationally' mean to you?

The research supports the training of future scientists that end up undertaking research in universities

- What do you understand by the fourth statement?

The research leads to the establishment of companies that create a small number of new jobs in the town where universities are based

- What do you understand by the fifth statement?
- What does 'a small number' mean to you?
- What type of jobs were you thinking about?
- What did you understand by 'the town where universities are based'?

The research produces a discovery that forms the basis of a start-up company that attracts small amounts of seed funding from investors [in exchange a small number of shares]

- What do you understand by the next statement?
- What type of discovery were you thinking of?
- What type of company were you thinking of?
- What do you understand by 'seed funding'?
- What type of investors were you thinking of?

Research contributes to an overseas company deciding to fund a follow-up study in the UK

- What do you understand by the next statement?
- What type of company were you thinking of?
- What do you understand by 'follow-up study'?

Research produces a discovery which is made freely available to companies and organisations and improves how they operate

- What do you understand by the next statement?
- What type of discovery were you thinking of?
- What types of companies and organisations were you thinking of?
- What types of improvements were you thinking of?
- What does 'freely available' mean to you?

Research contributes to an additional 6 months of life for a terminally ill patient free of disability

- What do you understand by this statement?

Findings from the research influence practice in the local hospital
{NO QUESTIONS, ALREADY COVERED IN SHEET 3}

Research contributes to a recommendation that a new treatment should entirely replace an existing treatment
{NO QUESTIONS, ALREADY COVERED IN SHEET 3}

Research contributes to better care being provided at very much higher cost
{NO QUESTIONS, ALREADY COVERED IN SHEET 3}

Researchers give interviews to the media about their research
{NO QUESTIONS, ALREADY COVERED IN SHEET 1}

Sheet 6

The research brings together the works of a range of others, identifying areas of consistency and difference
{NO QUESTIONS, ALREADY COVERED IN SHEET 2}

The research generates knowledge that is world-leading in terms of originality, significance and rigour
{NO QUESTIONS, ALREADY COVERED IN SHEET 1}

Research helps train scientists who go on to be recognised internationally in terms of originality, significance and rigour
{NO QUESTIONS, ALREADY COVERED IN SHEET 5}

The research supports the training of future scientists that end up working in the healthcare system
{NO QUESTIONS, ALREADY COVERED IN SHEET 2}

The research leads to the establishment of companies that create a substantial number of new jobs in regions where universities are based
{NO QUESTIONS, ALREADY COVERED IN SHEET 1}

The research produces a discovery that forms the basis of a start-up company that attracts small amounts of seed funding from investors [in exchange a small number of shares]
{NO QUESTIONS, ALREADY COVERED IN SHEET 5}

Research contributes to an overseas company deciding to set up a new UK research centre
{NO QUESTIONS, ALREADY COVERED IN SHEET 2}

Research produces a discovery which is the foundation of a new company
{NO QUESTIONS, ALREADY COVERED IN SHEET 1}

Research contributes to an additional 6 months of life for a terminally ill patient free of disability
{NO QUESTIONS, ALREADY COVERED IN SHEET 5}

Findings from the research influence practice in the local region
{NO QUESTIONS, ALREADY COVERED IN SHEET 4}

Research contributes to a recommendation that a new treatment should not be used
{NO QUESTIONS, ALREADY COVERED IN SHEET 4}

Research contributes to better care being provided at the same cost
{NO QUESTIONS, ALREADY COVERED IN SHEET 4}

Researchers talk in schools about their research
{NO QUESTIONS, ALREADY COVERED IN SHEET 3}

Wrap up

Thank you very much for taking the time to come in – it's been really helpful for us to hear your thoughts on the questions. As I mentioned at the start, we'll destroy the recording at the end of the study and your name won't be used in anything.

Are there any other questions you have now that we've finished?

Pilot

A pilot survey was conducted in November and December 2014. The MRC's grant holder database provided email addresses of 4708 researchers, out of which 500 were randomly selected to participate in the pilot. The pilot survey yielded 88 responses (complete and incomplete) from researchers and 100 complete responses from the general public. Specific questions asked about the respondent's understanding of the questionnaire and these confirmed that the questions were generally well understood by both groups and that respondents were engaging with the BWS experiment. Survey take-up by researchers was found to be a challenge. Hence the BWS experiment was simplified to reduce respondent burden and the MRC assisted in publicising the study through one of its official blogs and its Twitter account.
