# Supplementary material for: Understanding the relative valuation of research impact: a best–worst scaling experiment of the general public and biomedical and health researchers
Source: BMJ Open. 2016 Aug 18;6(8):e010916. doi: 10.1136/bmjopen-2015-010916 (PMC5013498; doi:10.1136/bmjopen-2015-010916)
Supplement: Supplementary file [file bmjopen-2015-010916supp4.pdf]

Supplementary File 4 – Survey questionnaires

GENERAL POPULATION QUESTIONNIARE
Part 0: Screening questions

Q1. Are you…?

1. Male
2. Female

Q2. How old are you?

Q3. Which UK region do you currently live in?

1. Channel Islands
2. East of England
3. East Midlands
4. London
5. North East
6. North West
7. Northern Ireland
8. Scotland
9. South East
10. South West
11. Wales
12. West Midlands
13. Yorkshire / Humberside
99. None of the above

Q4.

Please indicate the highest educational or professional qualification that you have obtained to date, if any:

1. GCSE/O Level/CSE
2. Vocational qualifications (=NVQ1+2)
3. A Level or equivalent (=NVQ3)
4. Bachelor degree or equivalent (=NVQ4)
5. Masters/PhD or equivalent
6. Other
7. No formal qualifications

Please indicate to which occupational group the Chief Income Earner in your household belongs, or which group fits best?

This could be you: the Chief Income Earner is the person in your household with the largest income. If the Chief Income Earner is retired and has an occupational pension please answer for their most recent occupation.

If the Chief Income Earner is not in paid employment but has been out of work for less than 6 months, please answer for their most recent occupation.

Semi or unskilled manual work (e.g. Manual workers, all apprentices to be skilled trades, Caretaker, Park keeper, non-HGV driver, shop assistant)	1
Skilled manual worker (e.g. Skilled Bricklayer, Carpenter, Plumber, Painter, Bus/ Ambulance Driver, HGV driver, AA patrolman, pub/bar worker, etc.)	2
Supervisory or clerical/ junior managerial/ professional/ administrative (e.g. Office worker, Student Doctor, Foreman with 25+ employees, salesperson, etc.)	3
Intermediate managerial/ professional/ administrative (e.g. Newly qualified (under 3 years) doctor, Solicitor, Board director small organisation, middle manager in large organisation, principal officer in civil service/local government)	4
Higher managerial/ professional/ administrative (e.g. Established doctor, Solicitor, Board Director in a large organisation (200+ employees, top level civil servant/public service employee))	5
Student	6
Casual worker – not in permanent employment	7
Housewife/ Homemaker	8
Retired and living on state pension	9
Unemployed or not working due to long-term sickness	10
Full-time carer of other household member	11
Other	98

Part 1: Introduction

We are a team of researchers from King's College London, RAND Europe and Cardiff University who are conducting a study of peoples' views on research. The study is funded by the UK Medical Research Council (MRC).

This sort of research is paid for by the government with tax payers' money, by charities such as the British Heart Foundation and by private companies, including pharmaceutical companies.

As part of this study, we are conducting the following survey which takes approximately 15 minutes to complete.

All responses will be anonymised and will be treated confidentially. Findings from combining all responses will be made publicly available via reports and research articles.

Should you require further information about this survey and our study, please click on this link http://www.kcl.ac.uk/sspp/policy-institute/projects/Innovation-Policy/Value-of-Impact.aspx.

Part 2: Best worst scaling experiment

The aim of the next part of the survey is to explore how different people value the different outcomes that can come from biomedical and health research.

Throughout this questionnaire we ask you to think about biomedical and health research that is conducted by people who work in universities. This sort of research is paid for by the government with tax payers' money, charities such as the British Heart Foundation and private companies, including pharmaceutical companies.

We shall now present you with eight ranking tasks.

Each task lists eight statements giving possible achievements of biomedical and health research. These achievements include examples of how research can help build knowledge, train future scientists, involve the public more closely in science, create jobs or other benefits for the economy, and improve people's health. In each of the eight tasks we will ask you to select research achievements, from those listed, which you think are most important, least important, second most important and second least important.

Please note that although some of the statements may be similar from one task to the next, there are often differences between the specific achievements being described and so it is important to read each set of statements carefully.

Example of a BWS Task

From the following list of potential contributions of biomedical and health research, which one do you feel is the **most** important?

Achievements of biomedical and health research	
1	Research reviews and combines previous findings, identifying areas of consistency and difference
2	Research trains young researchers who become doctors and nurses
3	Research contributes to the development of a treatment that would increase life expectancy by 1 year for the 10% of adults living with a common disease in the UK
4	Research generates knowledge that is recognised internationally
5	Research contributes to better care being provided at a higher cost
6	Research helps create a substantial number of new jobs across the UK
7	Researchers consult the public to help set research priorities
8	Research contributes to a company deciding to move a major part of its operations to the UK

Task 1 of 8

Now, we would like to know which of the remaining contributions is the **least** important.

Please note that the faded statement is the choice you previously made and cannot be selected again.

Task 1 of 8

Again, from the remaining contributions, please choose the one that you feel is the **most** important.

Please note that the faded statements are choices you previously made and cannot be selected again.

Task 1 of 8

Finally, from the remaining options, please choose the contribution that you feel is the **least** important.

Please note that the faded statements are choices you previously made and cannot be selected again.

Q5. In the ranking tasks, did you understand the descriptions?

1. Yes, all of them
2. Most of them
3. Some of them
4. No, not at all

Q6. Did you look at all the items presented in the ranking tasks?

1. Yes
2. No

Q7. Were you able to make comparisons and to a large extent informed choices?

1. Yes, in all tasks
2. In most of the tasks
3. In some of the tasks
4. No, not at all

Part 3: Public Attitudes to Science

Q8. How well informed do you feel, if at all, about science, and scientific research and developments?

1. Very well informed
2. Fairly well informed
3. Not very well informed
4. Not at all informed

Q9. For each of the following statements, could you indicate the extent to which you agree or disagree?

	Strongly agree	Tend to agree	Neither agree nor disagree	Tend to disagree	Strongly disagree	Don't know
The UK Government is working hard to ensure that people living in the UK will have enough fuel for our future needs						
Human activity does not have a significant effect on the climate						
People shouldn't tamper with nature						
I enjoy new situations and challenges						
God created the earth and all life in it						
It is important for me to keep on learning new skills						

Q10. Here are some statements about science. For each, please could you indicate the extent to which you agree or disagree?

	Strongly agree	Tend to agree	Neither agree nor disagree	Tend to disagree	Strongly disagree	Don't know
I don't understand the point of all the science being done today						
I see science and engineering differently						
Government funding for science should be cut because the money can be better spent elsewhere						
It is important to know about science in my daily life						
Science should be seen in isolation from other aspects of human knowledge						

Q11. Here are some statements about working in science. For each, please could you indicate the extent to which you agree or disagree?

	Strongly agree	Tend to agree	Neither agree nor disagree	Tend to disagree	Strongly disagree	Don't know
In general, scientists want to make life better for the average person						
Rules will not stop scientists doing what they want behind closed doors						
It is important to have some scientists who are not linked to businesses						
Government should delay the introduction of new medicines or technologies until scientists are completely certain there are no bad side effects						

Q12. Here are some statements about studying and working in science. For each, please could you indicate the extent to which you agree or disagree?
Please mark one option for each.

	Strongly agree	Tend to agree	Neither agree nor disagree	Tend to disagree	Strongly disagree	Don't know
Because of science and technology there will be more work opportunities for the next generation						
The science I learnt at school has been useful in my everyday life						
The UK needs to develop its science and technology sector in order to enhance its international competitiveness						
The maths I learnt at school has been useful in my job						

Q13. Here are some statements about how science is communicated and discussed. For each, please could you indicate the extent to which you agree or disagree? Please mark one option for each.

	Strongly agree	Tend to agree	Neither agree nor disagree	Tend to disagree	Strongly disagree	Don't know
Public consultation events are just public relations activities and don't make any difference to policy						
The Government should act in accordance with public concerns about science and technology						
We have no option but to trust those governing science >						
The public is sufficiently involved in decisions about science and technology						
Scientists should be rewarded for communicating their research to the public						
The media sensationalises science						

Part 4: Background questions [2]

Q14. Please choose the option that best describes your current work status:

1. Working full time (30 hours or more per week)
2. Working part time (less than 30 hours per week)
3. Looking after family or home
4. Full-time student
5. Retired
6. Not in paid employment because of long term illness or disability
7. Seeking work
8. Prefer not to say
9. Other [Please specify] [OPEN ANSWER]

Q15. Which, if any, of the following applies to you?
Please select all that apply

1. I have studied science to A Level or above
2. I am a scientist/researcher/academic
3. I am an engineer
4. I have scientists/researchers/academics among my friends
5. I have engineers among my friends
6. I have scientists/researchers/academics among my relatives
7. I have engineers among my relatives
8. I work with scientists/researchers/academics
9. I work with engineers

99. None of these

Q16. Which of the following groups do you consider you belong to?

1. **White:** British/Irish/Any other White background
2. **Mixed:** White and Black Caribbean/White and Black/White and Asian/Any other mixed/multiple ethnic background
3. **Asian or Asian British:** Indian/Pakistani/Bangladeshi/Any other West or South Asian background
4. **Black or Black British:** Caribbean/African/Any other Black background
5. **Arab**
6. Any other background (Please, specify)
7. Prefer not to say

Q17. What was your household income in the past 12 months from all sources, before tax and other deductions?

1. Under £10,399
2. £10,400 - £15,599
3. £15,600 - £20,799
4. £20,800 - £25,999
5. £26,000 - £31,199
6. £31,200 - £36,399
7. £36,400 - £41,599
8. £41,600 - £46,799
9. £46,800 - £51,999
10. £52,000 - £77,999
11. £78,000 - £103,999
12. £104,000 above
13. I prefer not to say

Part 5: Closing section

If you have any specific comments about the questions and how they were worded please share them with us in the box below.

If you have any further comments, please feel free to share those with us in the box provided below.

RESEARCHER QUESTIONNAIRE
Part 1: Introduction

We are a team of researchers from King's College London, RAND Europe and Cardiff University who are conducting a study of peoples' views on research. The study is funded by the UK Medical Research Council (MRC).

This sort of research is paid for by the government with tax payers' money, by charities such as the British Heart Foundation and by private companies, including pharmaceutical companies.

As part of this study, we are conducting the following survey which takes approximately 15 minutes to complete.

All responses will be anonymised and will be treated confidentially. Findings from combining all responses will be made publicly available via reports and research articles.

Should you require further information about this survey and our study, please contact us at research_impact@rand.org.

Part 2: Background questions [1]

Q1. What is your current job title?

If you hold multiple affiliations please use the title for the post for which you were awarded your most recent MRC grant.

1. Lecturer
2. Senior Lecturer
3. Reader
4. Professor
5. Research Assistant
6. Research Fellow
7. Senior Research Fellow
8. Research Professor
9. Other, please specify

Q2. What is the main type of research that you are involved in? (for definitions, please see http://www.hrcsonline.net/rac/summary)

Please select all that apply

1. Underpinning
2. Aetiology
3. Prevention
4. Detection and Diagnosis

5. Treatment Development
6. Treatment Evaluation
7. Disease Management
8. Health Services
9. Other <please specify>

Q3. What is your primary subject area? (please select all that apply)

1. Blood
2. Cancer
3. Cardiovascular
4. Congenital Disorders
5. Ear
6. Eye
7. Generic Health Relevance
8. Infection
9. Inflammatory and Immune System
10. Injuries and Accidents
11. Mental Health
12. Metabolic and Endocrine
13. Musculoskeletal
14. Neurological
15. Oral and Gastrointestinal
16. Renal and Urogenital
17. Reproductive Health and Childbirth
18. Respiratory
19. Skin
20. Stroke

98 Other

Q4. How many years have you worked as a researcher?

Q5. And of those, how many years have you been employed as a researcher in the UK?

Q6. Are you a practising clinician?

1. Yes
2. No

Q7. How many sessions of clinical practice do you have per week?

1. 1 session
2. 2 sessions
3. 3 sessions
4. 4 sessions
5. 5 sessions or more

Q8. Approximately how many journal articles have you published during the last 5 years?

Q9. Were you part of your university's 2014 REF submission?

1. Yes
2. No

Part 3: Best worst scaling experiment

We shall now present you with eight tasks.

Each task lists eight statements giving possible research impacts of biomedical and health research. These impacts include examples of how research can help build knowledge, train future scientists, involve the public more closely in science, create jobs or other benefits for the economy, and improve people’s health. While there are many more possible research impacts, they are out of scope for this study.
In each of the eight tasks we will ask you to select research impacts, from those listed, which you think are most important, least important, second most important and second least important.

Please note that although some of the statements may be similar from one task to the next, there are often differences between the specific impacts being described and so it is important to read each set of statements carefully.

Please note, you will not be able to go back once you select an answer so only click on a statement when you are sure.

We emphasise that this is an experimental approach so we would value your feedback once you have completed the survey. At the end of the survey there is a section to provide comments.

Example of a BWS Task

From the following list of potential contributions of biomedical and health research, which one do you feel is the **most** important?

Achievements of biomedical and health research	
1	Research reviews and combines previous findings, identifying areas of consistency and difference
2	Research trains young researchers who become doctors and nurses
3	Research contributes to the development of a treatment that would increase life expectancy by 1 year for the 10% of adults living with a common disease in the UK
4	Research generates knowledge that is recognised internationally
5	Research contributes to better care being provided at a higher cost
6	Research helps create a substantial number of new jobs across the UK
7	Researchers consult the public to help set research priorities
8	Research contributes to a company deciding to move a major part of its operations to the UK

Task 1 of 8

Now, we would like to know which of the remaining contributions is the **least** important.

Please note that the faded statement is the choice you previously made and cannot be selected again.

Task 1 of 8

Again, from the remaining contributions, please choose the one that you feel is the **most** important.

Please note that the faded statements are choices you previously made and cannot be selected again.

Task 1 of 8

Finally, from the remaining options, please choose the contribution that you feel is the **least** important.

Q10. In the ranking tasks, did you understand the descriptions?

1. Yes, all of them
2. Most of them
3. Some of them
4. No, not at all

Q11. Did you look at all the items presented in the ranking tasks?

1. Yes
2. No

Q12. Were you able to make comparisons and to a large extent informed choices?

1. Yes, in all tasks
2. In most of the tasks
3. In some of the tasks
4. No, not at all

**Part 4: Background questions [2]**

Q13. Are you…?

1. Male
2. Female

Q14. How old are you?

Q15. Please, indicate the highest educational or professional qualification that you have obtained to date?
Please tick all that apply.

1. Bachelor degree or equivalent
2. Medical Degree
3. Postgraduate diploma (PGDip) or certificate (PGCert)
4. Masters (MSc, MBA, MA) or equivalent
5. PhD or equivalent
6. Other

Q16. How many years has it been since the completion of your highest degree?

Q17. How many years have you spent in a country other than the UK in your professional career?

Q18. Please choose the option that best describes your current work status?

1. Working full time
2. Working part time

Q19. Which of the following groups do you consider you belong to?

1. White: British/Irish/Any other White background
2. Mixed: White and Black Caribbean/White and Black/White and Asian/Any other mixed/multiple ethnic background
3. Asian or Asian British: Indian/Pakistani/Bangladeshi/Any other West or South Asian background
4. Black or Black British: Caribbean/African/Any other Black background
5. Arab
6. Any other background [Please, specify]
7. Prefer not to say

Part 5: Closing section

Thank you for your participation in this survey. We are planning to disseminate the study findings widely. Would you be interested in receiving an email about the findings when the study is complete?

1. Yes
2. No

96. Prefer not to say

Please provide a contact email to receive the information about study findings. Note, your email will be stored separately from your response and will not be used for any other purpose.

Only King's College, RNAD Europe and Research Now will have access to this information which will be used solely for the purpose of sending you information about the study findings. This information will be destroyed after we have contacted you.

This survey questionnaire is now complete.
If you have any further comments, please feel free to share those with us in the box provided below.
