# Supplementary material for: Understanding the relative valuation of research impact: a best–worst scaling experiment of the general public and biomedical and health researchers
Source: BMJ Open. 2016 Aug 18;6(8):e010916. doi: 10.1136/bmjopen-2015-010916 (PMC5013498; doi:10.1136/bmjopen-2015-010916)
Supplement: Supplementary file [file bmjopen-2015-010916supp5.pdf]

Supplementary File 5: Additional information on the final model

Data quality checks

The robustness of the output from choice models can be improved by excluding data that are not valid (e.g. Hess et al., 2010). We conducted additional quality checks on the BWS data using three exclusion criteria. Firstly, we assumed that respondents who took less than 5 minutes to complete the survey were more likely to provide insincere answers. Secondly, we hypothesised that potentially ineligible data were generated by respondents who stated that they did not understand most choice tasks. Finally, respondents who stated that they were unable to make comparisons in most BWS tasks were also considered likely to provide unreliable answers. Table 3 in the main text shows the number of researcher and general population respondents who matched the above three criteria.

Preliminary model estimations using the full and reduced researcher sample data did not result in any significant improvement in model fit and therefore all 1171 researchers were included in modelling the BWS data. In contrast, for the general population, using the reduced data set (excluding the 272 respondents who met the criteria above), gave a significant improvement in model fit over the full sample. Therefore, the analysis of the BWS data in the general population sample was based on 728 respondents.

Respondent profiles in the general population and researcher samples

Tables SF5.1 and SF5.2 summarise respondent profiles in the researcher and general population samples, respectively. The MRC provided information about the distribution of researchers across age, gender and ethnicity, and we contrast these proportions against the researcher data used for modelling in Table SF5.1. The comparison focuses on the proportion of researchers with no missing data (966). In terms of gender, the two samples are significantly different with our survey over representing women (*p=0.01*). Also, our sample over represents White, Mixed and Black researchers and under represents Asian/Asian British researchers relative to the population of MRC-funded researchers (*p=0.001*). Finally, a smaller proportion of younger researchers is observed in our sample relative to the MRC database (note: a statistical test of independence is not possible due to a mismatch in the age categories).

Table SF5.1. Profile of respondents – Researchers

**Characteristics**	**Percentage of respondents (Percentages observed for all MRC grant holders)**
Gender (N= 966, completes only)	
Male	58.9 (63)
Female	41.1 (37)
Age (N=966, completes only)	
18 to 24	3.4 (up to 29 = 17.5)
25 to 34	17.7 (30 – 39 = 16.1)
35 to 44	26.2 (40 – 49 = 27.5)
45 to 54	26.9 (50 – 59 = 24.0)
55 to 64	19.6 (60 – 69 = 12.9)
65 and over	6.2 (70 and over = 1.9)
Ethnicity (N=966, completes only)	
White	86.9 (80.4)
Mixed	1.7 (1.5)

Asian/Asian British	3.7 (5.8)
Black/Black British	0.9 (0.6)
Other	2.7 (2.7)
Prefer not to say	4.1 (9.2)
Working Status (N=966, completes only)	
Full time	91.5
Part time	8.5
Education (N=966, completes only)	
PhD or equivalent	81.5
Medical degree	12.5
Practising clinician (N=1171, completes + incompletes)	20.1
Yes	79.9
No	
Job title (N=1170 completes + incompletes)	
Lecturer	6.9
Senior Lecturer	9.3
Reader	7.2
Professor/Emeritus professor	34.7
Research Assistant	1.7
Research Fellow	9.3
Senior Research Fellow	4.4
Research Professor	4.6
Clinical Fellow/Clinical Scientist9.3	1.4
PhD/DPhil Student	14.7
Group/Programme Leader/Head	3.1
Other	2.7
Experience as researcher in years (N=1171 completes + incompletes)	13.5
<=3	10.1
3 to 6	7.3
6 to 10	26.0
10 to 20	27.1
20 to 30	16.0
> 30	
Participated in 2014 REF (N=966, completes only)	
Yes	63.3
No	36.7
Years worked outside UK (N=966, completes only)	
0	45.7
1 to 5	35.9
6 to 10	11
11 to 20	5.6
Over 20	1.8

The general population sample was aimed at being representative against gender, age and region of residence based on mid-2013 ONS population estimates. Again, the comparison is conducted with respondents used in modelling the BWS. Our sample is representative of gender ($p = 0.145$) and region of residence ($p=0.294$), although it has been difficult to recruit respondents from Yorkshire and the Humber region (61 achieved vs. 80 target), hence additional respondents were sought from other northern regions including Scotland, North East and North West. Finally, our sample over represents individuals in the social grades ABC1 ($p=0.031$) and under represents respondents between the age of 18 and 24 years and over represents individuals aged 65 years and over ($p = 0.006$).

TableSF5.2. Profile of respondents – General Population

Characteristics (N = 728)	Percentage of respondents	ResearchNow targets used for setting the quota
Gender		
Male	46.3	49
Female	53.7	51
Age		
18 to 24	8.7	12
25 to 34	14.4	17
35 to 44	17.3	17
45 to 54	18.8	18
55 to 64	14.4	14
65 and over	26.4	22
Ethnicity		
White	93	
Mixed	1.2	
Asian/Asian British	2.2	
Black/Black British	1.4	Not used for targets
Arab	0.3	
Other	0.1	
Prefer not to say	1.8	
Working Status		
Full time	36.7	
Part time	16.5	Not used for targets
Looking after family / home	6.2	
Student	3.0	
Retired	27.8	
Other	9.1	
Prefer not to say	0.7	
NRS Social grade		
ABC1	55	51
C2DE	45	49
Education		
GCSE/O Level/CSE	19	
NVQ1+2 (Vocational	8.4	
qualification)	24.3	
NVQ3 (A level or equivalent)	29.5	
NVQ4 (Bachelor degree or	10.8	Not used for targets
equivalent)	2.2	
Masters/PhD or equivalent	5.8	
Other		
No formal qualification		
Region		
East	10.2	9
East Midlands	7.3	7
London	11.9	13
North East	4.3	4
North West	11.0	11
Northern Ireland	3.0	3
Scotland	8.5	8
South East	15.3	14
South West	8.5	9
Wales	5.1	5
West Midlands	10.0	9
Yorkshire Humberside	4.9	8
Income		
Under £10,399	6.9	Not used for targets
£10,400 - £15,599	9.9	

£15,600 - £20,799	9.9
£20,800 - £25,999	9.9
£26,000 - £31,199	8.6
£31,200 - £36,399	7.8
£36,400 - £41,599	6.6
£41,600 - £46,799	5.1
£46,800 - £51,999	5.8
£52,000 - £77,999	9.3
£78,000 - £103,999	5.2
£104,000 above	2.9
I prefer not to say	12.1

Segmentation

We also estimated models to test differences in preferences across segments within the general public and the researcher samples, respectively. Tests were conducted to identify segment-specific coefficients in the models. Only statistically significant differences were retained in the models reported in Table SF5.3. In the researchers' sample, the segments were defined based on research activity codes[1] which respondents provided as part of the survey questionnaire. Researchers who worked in more than one area were grouped within the 'Other' research category. The segments and corresponding number of researchers in the sample are shown in the following list:

1. Underpinning (327)
2. Aetiology (113)
3. Prevention (39)
4. Detection and Diagnosis (40)
5. Treatment Development (94)
6. Treatment Evaluation (27)
7. Disease Management (10)
8. Health Services (44)
9. Other + more than one research type (477)

In the general-population sample, each respondent was assigned to a segment based on their responses to a set of attitude statements taken from the 2014 Public Attitudes to Science (PAS) survey. This is the fifth survey in the PAS series commissioned by the Department for Business, Industry and Skills (and its predecessors[2]). The statements used were identified by Ipsos MORI in the most recent survey in the series (Castell et al., 2014) as a sub-set of all the statements in the survey that, taken together, can be used to allocate respondents to one of six attitudinal segments. The allocation of respondents to segments used the same methodology as that used by Ipsos MORI for the 2014 survey. (Personal Communication, 2014). The definitions of the segments obtained by Castell et al. (2014) and the corresponding numbers of respondents were as follows:

1. Confident Engagers (456): Individuals who "tend to have the most positive attitude towards science of all the segments, and have relatively few concerns about scientists, regulators, or the relationship between the Government and science. However, they are concerned about how the media reports science and the media's influence on science policy."

---

[1] http://www.hrcsonline.net/rac/summary

[2] The first survey was co-funded by The Wellcome Trust and published as *Science and the Public,* the Office of Science and Technology and The Wellcome Trust, October, 2000.

2. Distrustful Engagers (1): " highly enthusiastic about science but tend to be less trusting of scientists, regulators and the Government. Consequently, they tend to think the public should play a larger role in decision-making."
3. Late Adopters (191): "did not enjoy science at school, but have become more interested in it as adults, and now want to have more of a say in decision-making. Their interest tends to be linked to their environmental and ethical concerns, so they tend to be more engaged with specific issues such as climate change and genetically modified crops."
4. Concerned (2): "tend to have a more religious or spiritual outlook on life and consequently have stronger views on the limitations of science. They support Government efforts to consult the public on science, but have concerns about whether scientists themselves take the findings of these consultations on board."
5. Indifferent (9): "tend to be older, often retired people. They are not especially negative or worried about science, but tend to think science is not for people like them, so are less interested in finding out about it or in getting involved in decision-making."
6. Disengaged Sceptics (69): "have typically found science overwhelming since school, and do not feel informed about it today. They are often concerned about the speed of development in science, so tend to favour a conservative approach to regulation, and one that takes the public's views into account. However, they are less confident in getting involved themselves."

As there were very few respondents in some of the segments for both groups, it was not possible to identify statistically significant effects across all of the segments.

As shown in Table SF5.3, researchers who work in the area of “Health Services” value research-impacts related to the Cost domain higher than other segments. Also, those working in the area of “Underpinning” research value impacts related to the cost of providing care less than researchers working in other areas. Compared to researchers working in other areas, researchers working in the areas of “Underpinning” research also have a weaker preference for the research impact “Researchers consult the public to help set research priorities” but have a stronger preference for “Research contributes to a new UK research facility being set up by a company” and for all impacts in the Knowledge domain. Researchers working in “Prevention” value the life expectancy domain more than others.

In the general population segments we only identified small differences and they are difficult to coherently interpret. Specifically: Late Adopters place less value on the domains Dissemination and Training (for researchers, professors, doctors and nurses) than respondents belonging to any of the other five PAS segments. Late adopters along with Disengaged Sceptics place a higher value on the domain Impact (world leading, internationally excellent and recognised internationally) than the rest of the sampled respondents. Finally, Late Adopters and Confident Engagers value the Life Expectancy domain higher than the other segments in the sample.

Note that all coefficients in both models are estimated with respect to a reference research impact level set to zero (namely, “researchers give interviews to the media about their research” – DISS2). Hence all preferences are interpreted with respect to this reference.

Research Impact Valuation: Researchers and General Public

While coefficients presented in Table SF5.3 allow comparison within a sample (general public or researchers), the coefficients are not directly comparable between samples due to differences in

model scale between these two models, influenced by factors such as how well the respondents understood the task or how uniform the preferences are across the sample under consideration. This means that parameters representing identical preference structures but estimated from datasets with differing variance will differ in magnitude. The complication of the scale differences between two models can be avoided by computing the model coefficients as a ratio of two parameters.

The domain of life expectancy is specified as a continuous term (QOLYR) in the utility equation in order to capture the preference for each additional year of life expectancy. Further, a constant term – similar to an intercept in a linear model – (QOLYRC) is also specified for this domain which captures likely preference for this domain even when there is no improvement in life expectancy. Thus it is possible to convert all remaining coefficients into the unit of preference for each 'additional year of life expectancy for 10% of adults living with a common chronic disease in the UK' by dividing them with the coefficient of marginal increase in the life expectancy (QOLYR). For example, for the impact "Research contributes to a company deciding to move a major part of its operations to the UK" the coefficient is (2.10) in the researcher model, which can be expressed as a preference for (2.10/0.53 =) 3.94 additional years of life expectancy for 10% of adults living with a common chronic disease in the UK. In addition to facilitating comparison between groups, this ratio also converts the preferences from a dimensionless unit of utility to a more meaningful unit of additional years of life expectancy.

Table SF5.4 presents a preference value for each impact of research expressed in 'additional years of life expectancy'. For example, we find that the general public's preference for the impact JOBS1 ("Research helps create a small number of new jobs in the university") is equivalent to 3.72 [95% C.I.: 3.02, 4.41] additional years of life expectancy, whereas for researchers this preference is equivalent to 2.4 [95% C.I.: 1.95, 2.85] additional years of life expectancy. While the above transformation eliminates scale effects and allows cross-comparison between samples, it should be noted that the numbers (3.72 and 2.4) in both groups are ratios estimated to a reference level. Interpretation of these findings should be undertaken with a caution. A large ratio may therefore mean that the preference for a research impact corresponding to the numerator (e.g. JOBS1) is higher or that the preference for the research impact corresponding to the denominator (QOLYR) is lower, or both – with respect to the reference level (DISS2).

Table SF5.3. Profile of respondents – Researchers

Model group		General Public		Researchers	
**Description of research impact**	**Coeff. name**	**Coeff**	**95% CI**	**Coeff**	**95% CI**
Research contributes to care being provided more cheaply without any change in quality	COST1	4.469	[4.206, 4.732]	4.243	[4.001, 4.485]
Research contributes to better care being provided at the same cost	COST2	4.499	[4.237, 4.761]	4.793	[4.552, 5.034]
Research contributes to better care being provided at a higher cost	COST3	2.159	[1.91, 2.408]	2.703	[2.462, 2.943]
Research contributes to more choice of care at the same quality and cost	COST4	4.236	[3.975, 4.497]	3.312	[3.061, 3.563]
All impacts in domain "COST" * (Researcher segment = Health Services)	COST1234R8	n/a	n/a	0.845	[0.61, 1.08]
All impacts in domain "COST" * (Researcher segment = Underpinning)	COST1234R1	n/a	n/a	-0.543	[-0.668, -0.418]
Researchers talk in schools about their research	DISS1	0.893	[0.676, 1.11]	1.104	[0.882, 1.326]
Researchers give interviews to the media about their research	DISS2	0	n/a	0	n/a
Researchers give public lectures about their research	DISS3	0.31	[0.098, 0.521]	0.952	[0.731, 1.173]
Researchers consult the public to help set research priorities	DISS4	1.927	[1.686, 2.168]	2.263	[2.003, 2.523]
DISS4 * (Researcher segment = Underpinning)	DISS4R1	n/a	n/a	-1.577	[-1.916, -1.239]
All impacts in domain DISS * (General population segment = Late Adopters)	DISS1234S3	-0.506	[-0.679, -0.333]	n/a	n/a
Research generates knowledge that is world-leading	IMPACT1	3.362	[3.093, 3.631]	3.536	[3.295, 3.777]
Research generates knowledge that is internationally excellent	IMPACT2	4.174	[3.908, 4.441]	5.095	[4.857, 5.334]
Research generates knowledge that is recognised internationally	IMPACT3	3.607	[3.336, 3.877]	3.918	[3.678, 4.158]
Research generates knowledge that is recognised nationally	IMPACT4	3.591	[3.329, 3.854]	3.711	[3.471, 3.951]
All impacts in domain IMPACT * (General pop. segment = Disengaged Sceptics)	IMP1234S6	0.226	[0.03, 0.422]	n/a	n/a
All impacts in domain IMPACT * (General pop. segment = Late Adopters)	IMP123S3	0.275	[0.119, 0.431]	n/a	n/a
Research helps create a small number of new jobs in the university	JOBS1	1.524	[1.295, 1.753]	1.284	[1.064, 1.504]
Research helps create a small number of new jobs in one town	JOBS2	1.367	[1.139, 1.594]	0.153*	[-0.053, 0.358]
Research helps create a substantial number of new jobs in one region	JOBS3	1.712	[1.474, 1.95]	1.166	[0.949, 1.382]
Research helps create a substantial number of new jobs across the UK	JOBS4	3.23	[2.966, 3.493]	3.313	[3.077, 3.549]
Research replicates the work of others, helping to strengthen the evidence of how some things work	KNOW1	4.441	[4.175, 4.707]	5.463	[5.222, 5.704]
Research results in a new finding, helping to focus subsequent research activities	KNOW2	2.5	[2.247, 2.753]	3.345	[3.099, 3.59]
Research shows that something does not work, eliminating the need for further investigation	KNOW3	3.922	[3.653, 4.19]	5.028	[4.786, 5.27]
Research reviews and combines previous findings, identifying areas of consistency and difference	KNOW4	3.406	[3.142, 3.67]	2.806	[2.552, 3.059]
All impacts in domain "KNOW" * (Researcher segment = Underpinning)	KNOW1234R1	n/a	n/a	0.386	[0.282, 0.489]
Research contributes to a follow-up study in the UK being funded by a company	PVT1	2.688	[2.437, 2.939]	1.067	[0.851, 1.283]
Research contributes to an existing UK research facility being partly funded by a company	PVT2	2.677	[2.427, 2.928]	1.084	[0.866, 1.303]
Research contributes to a new UK research facility being set up by a company	PVT3	2.679	[2.425, 2.933]	1.512	[1.272, 1.752]
Research contributes to a company deciding to move a major part of its operations to the UK	PVT4	2.852	[2.591, 3.112]	2.133	[1.893, 2.374]
PVT3 * (Researcher segment = Underpinning)	PVT3R1	n/a	n/a	0.719	[0.408, 1.03]
Research trains young researchers who become researchers in industry	TRAIN1	3.674	[3.41, 3.937]	3.124	[2.889, 3.359]
Research trains young researchers who become university professors	TRAIN2	3.328	[3.066, 3.59]	3.817	[3.58, 4.053]
Research trains young researchers who become doctors and nurses	TRAIN3	3.737	[3.47, 4.003]	2.874	[2.634, 3.113]
Research trains young researchers who go on to work outside of science	TRAIN4	2.331	[2.076, 2.586]	2.13	[1.889, 2.37]
First three TRAIN impacts * (General pop. segment = Late Adopters)	TRN123S3	-0.308	[-0.479, -0.138]	n/a	n/a
Value of change in 1 year on life expectancy of 10% of adults living with a common chronic disease in the UK	QOLYR	0.41	[0.365, 0.455]	0.535	[0.496, 0.574]
Intercept on life expectancy	QOLYRC	3.942	[3.644, 4.239]	3.986	[3.751, 4.22]
QOLYRC * (Researcher segment = Prevention)	QOLYRCR3	n/a	n/a	0.635	[0.404, 0.866]
QOLYRC * (General pop. segment = Late Adopters)	QOLYRCS3	0.421	[0.236, 0.606]	n/a	n/a
QOLYRC * (General pop. segment = Confident Engagers)	QOLYRCS1	0.367	[0.2, 0.534]	n/a	n/a
Impact statement position - bottom most	Bottom	0.128	[0.032, 0.224]	0.398	[0.297, 0.498]
Impact statement position - second from the top	Top2	0.145	[0.077, 0.212]	0	n/a
Impact statement position - top most	Top	0.192	[0.126, 0.259]	0.171	[0.113, 0.228]
Scale for second worst preference	Scale4	0.468	[0.437, 0.499]	0.361	[0.339, 0.384]
Scale for second best preference	Scale3	0.621	[0.584, 0.657]	0.578	[0.553, 0.603]
Scale for worst preference	Scale2	0.591	[0.555, 0.627]	0.483	[0.456, 0.51]
Scale for best preference(fixed to one)	Scale1	1	n/a	1	n/a
* p = 0.138					

Table SF5.4. Research impact weights with segmentation expressed in units of life expectancy

		General Public					Researchers				
Model group		PAS segments				All Segments	Research area segment				All Segments
Description of research impact	Coeff. name	Confident Engagers	Late Adopters	Disengaged Sceptics	Others		Underpinning	Health Services	Prevention	Others	
Research contributes to care being provided more cheaply without any change in quality	COST1	-	-	-	-	10.9 [9.54, 12.27]	6.92 [6.23, 7.6]	9.51 [8.59, 10.43]	-	7.93 [7.2, 8.66]	n/a
Research contributes to better care being provided at the same cost	COST2	-	-	-	-	10.98 [9.61, 12.34]	7.94 [7.21, 8.68]	10.54 [9.57, 11.51]	-	8.96 [8.18, 9.74]	n/a
Research contributes to better care being provided at a higher cost	COST3	-	-	-	-	5.27 [4.42, 6.11]	4.04 [3.48, 4.59]	6.63 [5.85, 7.41]	-	5.05 [4.47, 5.63]	n/a
Research contributes to more choice of care at the same quality and cost	COST4	-	-	-	-	10.34 [9.04, 11.64]	5.17 [4.56, 5.79]	7.77 [6.93, 8.61]	-	6.19 [5.54, 6.84]	n/a
Researchers talk in schools about their	DISS1	-	0.94 [0.28, 1.61]	-	2.18 [1.6, 2.76]	n/a	-	-	-	-	2.06 [1.62, 2.5]
Researchers give interviews to the media about their research	DISS2	-	-1.24 [-1.68, -0.79]	-	-	n/a	-	-	-	-	n/a
Researchers give public lectures about their	DISS3	-	-0.48 [-1.14, 0.18]	-	0.76 [0.23, 1.28]	n/a	-	-	-	-	1.78 [1.35, 2.21]
Researchers consult the public to help set research priorities	DISS4	-	3.47 [2.68, 4.25]	-	4.7 [3.92, 5.49]	n/a	1.28 [0.67, 1.9]	-	-	4.23 [3.65, 4.81]	n/a
Research generates knowledge that is world-	IMPACT1	-	8.87 [7.66, 10.08]	8.75 [7.51, 10]	8.2 [7.08, 9.32]	n/a	-	-	-	-	6.61 [5.95, 7.27]
Research generates knowledge that is internationally excellent	IMPACT2	-	10.85 [9.47, 12.24]	10.74 [9.33, 12.15]	10.18 [8.89, 11.48]	n/a	-	-	-	-	9.52 [8.71, 10.33]
Research generates knowledge that is recognised internationally	IMPACT3	-	9.47 [8.21, 10.73]	9.35 [8.06, 10.64]	8.8 [7.63, 9.97]	n/a	-	-	-	-	7.32 [6.63, 8.02]
Research generates knowledge that is recognised nationally	IMPACT4	-	-	9.31 [8.03, 10.6]	8.76 [7.6, 9.92]	n/a	-	-	-	-	6.93 [6.26, 7.61]
Research helps create a small number of new jobs in the university	JOBS1	-	-	-	-	3.72 [3.02, 4.41]	-	-	-	-	2.4 [1.95, 2.85]
Research helps create a small number of new jobs in one town	JOBS2	-	-	-	-	3.34 [2.67, 4]	-	-	-	-	0.29 [-0.1, 0.67]
Research helps create a substantial number of new jobs in one region	JOBS3	-	-	-	-	4.18 [3.43, 4.92]	-	-	-	-	2.18 [1.74, 2.61]
Research helps create a substantial number of new jobs across the UK	JOBS4	-	-	-	-	7.88 [6.8, 8.96]	-	-	-	-	6.19 [5.56, 6.82]
Research replicates the work of others, helping to strengthen the evidence of how	KNOW1	-	-	-	-	10.84 [9.48, 12.19]	10.93 [10.02, 11.84]	-	-	10.21 [9.36, 11.07]	n/a
Research results in a new finding, helping to focus subsequent research activities	KNOW2	-	-	-	-	6.1 [5.18, 7.02]	6.97 [6.28, 7.67]	-	-	6.25 [5.61, 6.9]	n/a
Research shows that something does not work, eliminating the need for further	KNOW3	-	-	-	-	9.57 [8.33, 10.81]	10.12 [9.26, 10.98]	-	-	9.4 [8.59, 10.2]	n/a
Research reviews and combines previous findings, identifying areas of consistency	KNOW4	-	-	-	-	8.31 [7.19, 9.43]	5.96 [5.31, 6.62]	-	-	5.24 [4.63, 5.85]	n/a
Research contributes to a follow-up study in the UK being funded by a company	PVT1	-	-	-	-	6.56 [5.61, 7.51]	-	-	-	-	1.99 [1.56, 2.42]
Research contributes to an existing UK research facility being partly funded by a	PVT2	-	-	-	-	6.53 [5.59, 7.48]	-	-	-	-	2.03 [1.59, 2.46]
Research contributes to a new UK research facility being set up by a company	PVT3	-	-	-	-	6.54 [5.58, 7.49]	4.17 [3.5, 4.84]	-	-	2.83 [2.33, 3.32]	n/a
Research contributes to a company deciding to move a major part of its operations to the	PVT4	-	-	-	-	6.96 [5.96, 7.96]	-	-	-	-	3.99 [3.45, 4.52]
Research trains young researchers who become researchers in industry	TRAIN1	-	8.21 [7.07, 9.36]	-	8.96 [7.78, 10.14]	n/a	-	-	-	-	5.84 [5.23, 6.45]
Research trains young researchers who become university professors	TRAIN2	-	7.37 [6.3, 8.44]	-	8.12 [7.02, 9.22]	n/a	-	-	-	-	7.13 [6.46, 7.81]
Research trains young researchers who become doctors and nurses	TRAIN3	-	8.36 [7.2, 9.53]	-	9.12 [7.92, 10.32]	n/a	-	-	-	-	5.37 [4.78, 5.96]
Research trains young researchers who go on to work outside of science	TRAIN4	-	-	-	5.69 [4.8, 6.57]	n/a	-	-	-	-	3.98 [3.45, 4.52]
Intercept on life expectancy	QOLYRC	10.512 [9.083, 11.942]	10.64 [9.19, 12.1]	-	9.62 [8.22, 11.01]	n/a	-	-	8.64 [7.71, 9.56]	7.45 [6.69, 8.21]	n/a
	Note:	'-' is displayed when the a segment is merged with others									

References

Hess S, Rose JM, Polak J. Non-trading, lexicographic and inconsistent behaviour in stated choice data. *Transportation Research Part D* 2010; 15, 405–417
